# Climate-driven diversification in two widespread *Galerida *larks

**DOI:** 10.1186/1471-2148-8-32

**Published:** 2008-01-29

**Authors:** Alban Guillaumet, Pierre-André Crochet, Jean-Marc Pons

**Affiliations:** 1 Institut des Sciences de l'Evolution, C.C. 63, Université de Montpellier II, Place E. BATAILLON, 34095 Montpellier Cedex, France; 2 EPHE – UMR 5175, Centre d'Ecologie Fonctionnelle et Evolutive, 1919 route de Mende, 34293 Montpellier cedex 5, France; 3 CNRS – UMR 5175, Centre d'Ecologie Fonctionnelle et Evolutive, 1919 route de Mende, 34293 Montpellier cedex 5, France; 4 Origine, Structure et Evolution de la biodiversité, UMR 5202, C.P. 51, 55 rue Buffon, 75005 Paris, France; 5 Service de Systématique moléculaire, IFR 101 CNRS, 43 rue Cuvier, 75005 Paris, France

## Abstract

**Background:**

The major impact of Plio-Pleistocene climatic oscillations on the current genetic structure of many species is widely recognised but their importance in driving speciation remains a matter of controversies. In addition, since most studies focused on Europe and North America, the influence of many other biogeographic barriers such as the Sahara remains poorly understood. In this paper, climate-driven diversification was investigated by using a comparative phylogeographic approach in combination with phenotypic data in two avian species groups distributed on both sides of the deserts belt of Africa and Asia. In particular, we tested whether: 1) vicariance diversification events are concomitant with past climatic events; and 2) current ecological factors (using climate and competition as proxies) contribute to phenotypic divergence between allopatric populations.

**Results:**

Mitochondrial and nuclear sequence data indicated that the crested and Thekla lark species groups diverged in the early Pliocene and that subsequent speciation events were congruent with major late Pliocene and Pleistocene climatic events. In particular, steep increase in aridity in Africa near 2.8 and 1.7 million years ago were coincident with two north-south vicariance speciation events mediated by the Sahara. Subsequent glacial cycles of the last million years seem to have shaped patterns of genetic variation within the two widespread species (*G. cristata *and *G. theklae*). The Sahara appears to have allowed dispersal from the tropical areas during climatic optima but to have isolated populations north and south of it during more arid phases. Phenotypic variation did not correlate with the history of populations, but was strongly influenced by current ecological conditions. In particular, our results suggested that (i) desert-adapted plumage evolved at least three times and (ii) variation in body size was mainly driven by interspecific competition, but the response to competition was stronger in more arid areas.

**Conclusion:**

Climatic fluctuations of the Plio-Pleistocene strongly impacted diversification patterns in the *Galerida *larks. Firstly, we found that cladogenesis coincides with major climatic changes, and the Sahara appears to have played a key role in driving speciation events. Secondly, we found that morphology and plumage were strongly determined by ecological factors (interspecific competition, climate) following vicariance.

## Background

All models of speciation implicitly recognize two steps, whereby initial divergence is followed by evolution of reproductive isolation. In the standard allopatric model (in contrast to ecological speciation models), geographical isolation is viewed as a prerequisite for divergence [1]. While geographical isolation may be due to dispersal or vicariance (e.g. tectonics), the refuge theory argues that climate change is generally responsible for the fragmentation of a species range [2]. For populations that remain isolated a long time, climate-driven vicariance events may be sufficient for reproductive isolation to evolve by drift alone [3]. Nevertheless, adaptation to local ecological conditions in different refuges can also accelerate speciation when divergence affects phenotypic traits involved in mate choice [1,4-6]. Accordingly, climatic fluctuations of the Plio-Pleistocene (succession of glacial and interglacial events) may be expected to produce a stasis-punctuation model of evolution, where bursts of speciation are caused by major climatic changes [7-10] (hereafter, the climate-driven speciation hypothesis, from which the much-debated "LPO" Late Pleistocene Origin model [11] is a derivative).

However, while there is a consensus around the idea of past climatic oscillations having been determinant in shaping the current distribution range and genetic structure of many modern animal and plant species [12,13], their importance in driving speciation remains a matter of controversies [14-16], a situation that results from several factors. Firstly, the timing of speciation events is often inaccurate or too imprecise [17]. Secondly, despite an expanding literature on the role of ecology in driving the patterns of phenotypic differentiation and the levels of gene flow between sympatric or parapatric populations, the relationship between ecology and allopatric speciation remains poorly understood [18]. Thirdly, recent analyses suggested that the importance of the Pleistocene for speciation events might actually vary among regions [16] and habitats [19,20]. However, the majority of studies focused on European and North American species, even if climatic cycles were as pronounced elsewhere [21,22].

In Africa for instance, frequent climatic shifts of moderate amplitude before 2.8 million years ago (MYA) were followed by three major steplike shifts towards more arid conditions near 2.8 (± 0.2), 1.7 (± 0.1) and 1.0 (± 0.2) MYA. Then, the climate changed quite abruptly around 0.9 MYA with an accentuation of amplitude and duration of climatic cycles (development of 100 KY late Pleistocene glacial climatic cycles [21,23]). Periods of increased aridity were characterized by a contraction of forests and an expansion of savannas and deserts [21], and this have affected the biotas that occupy these biomes. Fossil African bovid and rodent assemblages shifted towards arid-adapted species between 2.7 and 2.5 MYA, and a further increase in the relative abundance of arid-adapted bovid species was noticed in Africa near both 1.7 and 1.0 MYA [21,23]. Also, these periods correspond to key junctures in early hominid evolution, including the emergence of the genus *Homo *[23]. In addition, some recent phylogeographical studies in Africa revealed a good congruence between divergence times among species or phylogroups and the peak of aridification that occurred 1 MYA ([24] for the African greenbul *Andropades tephrolaemus*, [25] for the African montane forest robin *Pogonocichla stellata *and [26] for the phytophageous insect *Busseola fusca*). Furthermore, several intraspecific radiations appear to have occurred within the past 200–300 KY, corresponding to the last two or three major Pleistocene glaciations (e.g. in the hartebeest *Alcelaphus buselaphus *[7] and in the Akalats of the genus *Shepphardia *[27]).

However, generality of these results might be affected by biases in the current sampling of groups and biomes, with savanna-dwelling mammals [7,22,28-31] and forest avian species [24,25,32] being over represented in comparison with arid regions which are somewhat neglected. As a consequence the role of the Sahara in driving speciation events is still poorly understood [33,34], while we may expect that periods of increased aridity, that actually started more than 3 million years ago [35] and corresponded to an expansion of the Sahara, promoted north-south vicariance events [36,37]. Since climatic fluctuations appeared to affect arid regions in the Arabian peninsula and south-east Asia (e.g. Thar desert in NE India) in a similar fashion (i.e. glacial periods were associated with desert expansion [38-40]), it is further possible that Plio-Pleistocene climatic fluctuations were responsible for additional east-west (e.g. sub-Saharan Africa-India) vicariance events.

In this paper, we investigated the applicability of the climate-driven speciation hypothesis to avian populations distributed around the desert belt in North Africa and Eurasia. In particular, we tested whether (i) vicariance events were concomitant with Plio-Pleistocene climatic changes and (ii) current ecological factors (using climate and competition as proxies) contribute to phenotypic divergence between allopatric populations. We choose the crested and Thekla lark species complexes as model because: (i) they have a very large Old World distribution north and south of the Sahara in Africa, but also to the east within and beyond the arid belt of Asia; (ii) they show a high level of phenotypic diversification, resulting in a large number of described subspecies [41]; (iii) the crested and the Thekla lark species group have diverged ~3.7 MYA [42], and thus represent two independent evolutionary trajectories through Plio-Pleistocene climatic fluctuations. In addition, they are species of open grounds (semi-desert, savanna, highland steppes), for which relevant phylogeographic data in Africa are scarce, particularly in comparison with mammals and forest birds (see above; but see [43]).

**Figure 1:**
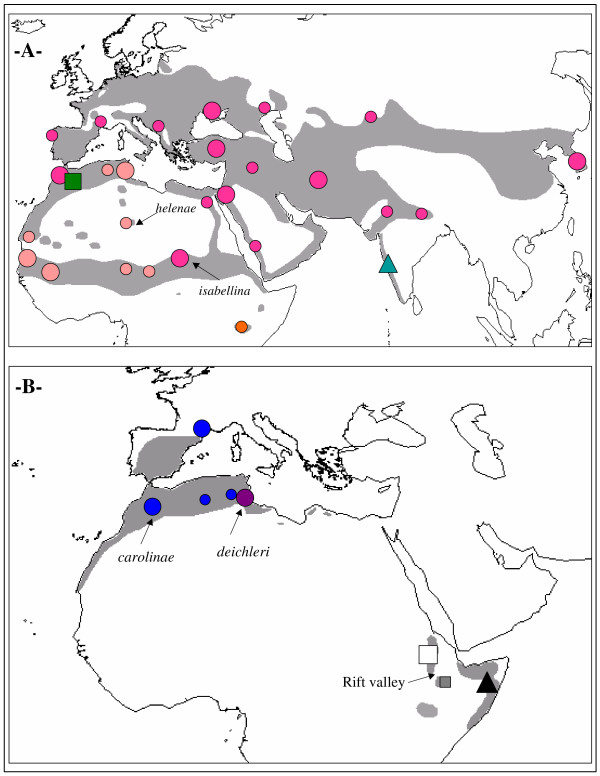
**Phylogeography**. Geographic distribution of the short -291 bp- cytochrome b fragment. Symbol size accounts for the number of specimens: n = 1 for small symbols, n ≥ 2 otherwise (see Tables 4–5 for exact values). 1a) crested lark: *G. randonii *= square (see [42] for details on its Moroccan distribution); *G. malabarica *= triangle; *G. cristata *= circles (*cristata *= red; *senegallensis *= salmon; *somaliensis *= orange). Whenever possible, specimens in different localities within a single country are represented separately (Chad, Algeria, Russia). 1b) Thekla lark: *G. theklae *= circle (*theklae *= blue; *superflua *= purple; in Tunisia, the westernmost specimen with a *theklae *haplotype is shown separately); *ellioti *= triangle; *praetermissa *= white square; *hueii *= grey square (the Rift valley separating *praetermissa *and *hueii *in Ethiopia is indicated). We also show the geographic position of the sandy subspecies used to test the hypothesis of convergent evolution of plumage patterns (*helenae *and *isabellina *for the crested lark; *carolinae *and *deichleri *for the Thekla lark).

Prior analyses in the crested lark complex revealed the existence of two reproductively isolated and essentially allopatric species (*G. cristata *and *G. randonii*). *Galerida randonii *is an endemic of central Maghreb while *G. cristata *has a much wider distribution [44] (Fig. 1a). Limited geographic sampling further revealed the existence of two distinct genetic groups in *G. cristata*: *cristata *has the largest distribution from western Morocco to the Middle East and through Eurasia, and possibly also in eastern Sahel. The *senegallensis *group was found on both sides of the Saharan desert, in the western Sahel and in the eastern Maghreb [44].

The distribution of the Thekla lark is disjoint, with one set of populations in the western Mediterranean region, and the other one in the Horn of Africa (Fig. 1b). In addition, *G. malabarica *of coastal India might form a superspecies with the Thekla lark [41]. Since these three sets of populations are currently separated by Arabo-Saharan deserts, we may speculate that they became isolated following one or several episodes of increased aridity in these areas (see above). However, a single study has examined genetic variation in *G. theklae*, but it was limited to Morocco and did not detect any phylogeographical structure among five described subspecies [42]. In this study, important new sampling (mostly museum specimens, including *G. malabarica*) and new analyses (phylogenetic dating, population genetics) were used to investigate the evolutionary history of modern taxa belonging to the crested and Thekla lark species complexes and its links with past climatic events.

In addition to examining historical patterns of diversification events in these two partially co-distributed species complexes, phylogenetic and population genetics data were used to understand the processes which underlie complex patterns of phenotypic differentiation in these taxa. In particular, we tested the hypothesis that recurrent adaptation to similar living environments provides a tenet for long-standing systematic controversies [41]. By studying traits (e.g. body size, bill shape and coloration) that are prominent in mate choice in passerine birds [5,45], this study also brought insights into speciation patterns in these taxa and biogeographic zones.

Firstly, previous analyses in the crested lark complex revealed a striking syndrome of body size increase in the Maghreb for two different lineages [44]. However, previous studies did not make further attempts to explain this pattern, which could be explained either by climatic effect (e.g. James' version of Bergmann's rule, which predicts larger body size in more arid areas [46]), and/or by interspecific competition (i.e. character displacement in the Maghreb where crested larks live sympatrically with the Thekla lark). The design of the present study allowed us to address the climatic component of the variation (by testing the repeatability of this pattern in the Thekla lark complex) while simultaneously controlling for the possible influence of interspecific competition (by including both allopatric and sympatric populations) and phylogenetic effects (by using a comparative method).

Secondly, subspecies that live in desertic areas tend to have a very pale sandy plumage, seemingly mainly as a result of drastic reduction in the amount of melanin deposition in feathers centre of the head, back, wings and breast [41] (pers. obs.). However, such phenotypic similarity might reflect close phylogenetic relationships rather than independent adaptation to desert conditions. For instance, no mtDNA divergence was found between two desert subspecies of the Thekla lark (*aguirrei *and *carolinae*) in a previous study in Morocco [42]. In this study, phylogenetic differentiation of desert subspecies was investigated for geographically remote populations, both in the Thekla lark complex (*deichleri *of S Tunisia *versus carolinae *of the Figuig area in Morocco) and in the crested complex (*helenae *of Tassili in Algeria *versus isabellina *of Ennedi, Chad; see Fig. 1 for locations and Additional file 2 for illustrations). In addition, a coalescence-based method was used to investigate the possible influence of gene flow in establishing similarity between desert subspecies.

In summary, this paper addresses the influence of the climate on speciation and phenotypic variation in two widespread species complexes of larks. Incidentally, this study should also bring new insights into the biogeography of poorly-known regions such as the Sahara, and to the phylogenetic status of currently isolated populations in India or the Horn of Africa.

## Results

### Phylogeny

**Figure 2:**
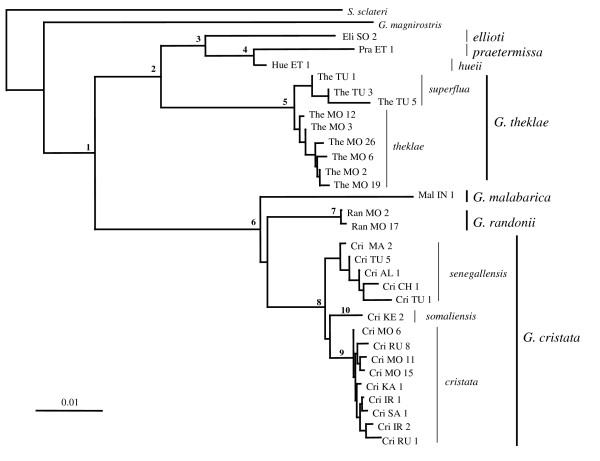
**MtDNA Phylogeny**. The topology was obtained for the whole data set using NJ and the pairwise gap removal option (average length = 665 bp); bootstrap support and divergence time for supported nodes (at least one bootstrap value > 70) are given in Table 1. Note that the very short branch of *hueii *as compared to *praetermissa *is partly due to the fact that we could not obtain the whole H'F cyt b fragment (i.e. the most variable fragment) for *hueii*.

As expected, two major groups broadly corresponding to the Thekla lark complex on one hand, and to the crested lark complex on the other hand were identified: for nuclear data, 6–7 mutations were fixed between the two species complexes, as compared to 0–1 mutation among sequences within groups, and for mtDNA, both groups were found to be monophyletic with strong bootstrap support (BS ≥ 94; Fig. 2; Table 1). However, despite its Thekla-like morphology, *G. malabarica *turned out to be a member of the crested lark complex. This result is supported by identical mtDNA sequences of three different specimens of *G. malabarica *(two of them are included in the 291 bp alignment; see Table 2, while the third one had a sequence of 244 bp), and by the 115 bp of the β-fibrinogen gene obtained for four different *G. malabarica *(Table 3): the four specimens shared three mutations that are diagnostic of crested larks (sites 7, 66 and 82), but they were not fixed for any mutation diagnostic of the Thekla lark complex (even if they were polymorphic for one mutation – at site 20 – that is otherwise diagnostic of the Thekla lark complex).

**Table 1:** Divergence times

Node	Divergence event	Bootstrap	Divergence time 2%/MY	Climatic event	Divergence time 1.5%/MY	Divergence time 2.5%/MY
1	species of crested/sp. of Thekla	83, 92, 93	4.96 [3.86–6.09]	/	6.61 [5.15–8.12]	3.97 [3.09–4.87]
2	Ethiopian/Palearctic Thekla	94, 98,100	2.81 [2.11–3.56]	Aridification 2.8 ± 0.2	3.75 [2.81–4.75]	2.25 [1.69–2.85]
3	*ellioti*/*praetermissa*	84, 69, 82	1.83 [1.23–2.43]	Aridification 1.7 ± 0.1	2.44 [1.64–3.24]	1.46 [0.98–1.94]
4	*hueii*/*praetermissa*	82,82,80	1.08 [0.55–1.65]	Aridification 1.0 ± 0.2	1.44 [0.73–2.20]	0.86 [0.44–1.32]
5	Div. within G. theklae *	98,100,100	0.15 [0.03–?]	Glacial cycles < 0.9	0.20 [0.04–?]	0.12 [0.02–?]
6	Div. 3 species crested larks	100,100,98	1.77 [1.15–2.46]	Aridification 1.7 ± 0.1	2.36 [1.53–3.28]	1.42 [0.92–1.97]
7	Div. within *G. randonii*	100,99,93	/	/	/	/
8	Div. within G. cristata *	99,89,75	0.39 [0.07– ?]	Glacial cycles < 0.9 MYA	0.52 [0.09–?]	0.31 [0.06–?]
9	Div. within *G. c. cristata*	93,80,80	/	/	/	/
10	Div. within *G. c. somaliensis*	85,96,94	/	/	/	/

Bootstrap support (NJ, MP, ML) and estimates of divergence times in million years ago (using BEAST or MDIV – latter indicated with * -) derived for various nodes of the mitochondrial phylogeny (shown in Fig. 2) using the standard molecular clock of 2% per million year [95% credibility interval]. Possible coincidence with some climatic events in MYA (after [21]) is indicated, as well as modified estimates derived from slightly different molecular clocks (1.5 to 2.5%/MY; see additional file 1b for further details).

**Table 2:** Variable DNA sites matrix (cytochrome b)

		1111111111111112222222222222222222	
		112233457888990012445556788990000111122234566777	
Haplotype	GenBank	17032517350589143927382343217061268136806901925479	n
The_MO_2	AY769740	TCCTCCTACTACACTACGATCCGCCTATAGATCCCGTTACCACACATCCT	31
The_MO_6	AY769742	..T...............................................	1
The_MO_12	AY769743	.................A................................	1
The_MO_19	AY769741	...............................C..................	4
The_TU_1	EF445418	.........................C.................C.G....	1
The_TU_3	EF445419	.........................C.................C......	4
Pra_ET_1	EF445421	.....TC....A..C....C..A......A....TA...T..TC.G..T.	2
Eli_SO_2	EF445423	...CTTC....A..C.TAC..TA...G..A.....A......TC.G...C	2
Cri_MO_6	AY769746	CT.C..CG.CC.GTC..AC...ATGC.C..G....A.C...G.CTGCT..	51
Cri_MO_11	AY769748	CT.C..CG.CC.GTC..AC...ATGC.C..G....AAC...G.CTGCT..	1
Cri_IR_2	DQ028951	CT.C..CG.CC.GTC..AC...ATGC.C..G..T.A.CG..G.CTGCT..	1
Cri_RU_1	EF445426	CT.C..CG.CC.GTC..AC...ATGC.C..G..T.A.C...G.CTGCT..	2
Cri_RU_8	EF445427	CT.C..CGTCC.GTC..AC...ATGC.C..G....A.C...G.CTGCT..	3
Cri_AL_1	DQ028953	CT.C..CG.CC...C..AC...ATGC.C..G....A.C...G.TT.CT..	12
Cri_CH_1	DQ028954	CT.C..CG.CC.C.C..AC...ATGC.C..G....A.C...G.TT.CT..	1
Cri_MA_2	DQ028956	CT.C..CG.CC...C..AC...ATGC.C..G....A.C.....TT.CT..	1
Cri_TU_1	DQ028955	CT.C..CG.CC...C..AC...ATGC.C..G....A.C..AG.TT.CT..	3
Cri_KE_2	EF445429	CT.C..CG.CC...C..AC...ATGC.C..G..T.A.C...G.TTGCT..	1
Ran_MO_2	AY769749	.T.C..CG.CC..TCG.AC...ATG..C.......A.C.....TTGCT.C	19
Mal_IN_1	EF445430	CT.C.TCG.CC...C..AC.T.ATG..CGAG.A....C.....TTGCT.C	2

20 haplotypes for the short (291 bp) cytochrome b fragment (see Tables 4–5 for the geographic origin of samples).

**Table 3:** Variable DNA sites matrix (β-fibrinogen)

		222		
		12689223		
	GenBank	740626390	n	Species (geography)
T1	AY769756	GAGCGTCCT	12	*G. theklae *(Mo:6, Tu:3), *G. ellioti *(1), *G. praetermissa *(2)
T2	EF445416	.G.......	1	*G. ellioti *(1)
H	AY769759	A.CAA.TAG	19	*G. randonii *(6), *G. cristata *(Mo:6,Tu:2,Ch:2,Se:1,Ke:1,Ir:1)
M1	/	A.SAAYNNN	2	*G. malabarica *(2)
M2	EF445417	A.GAACNNN	2	*G. malabarica *(2)

5 alleles (or genotypes) for Intron 7 of the β-fibrinogen gene (for the two *G. malabarica *called M1, the phase of the alleles was not unravelled); *S *= C and G; *Y *= C and T. The geographic origin of samples is indicated as follows: Mo = Morocco; Tu = Tunisia; Ch = Chad; Se = Senegal; Ke = Kenya; Ir = Iran.

**Figure 3:**
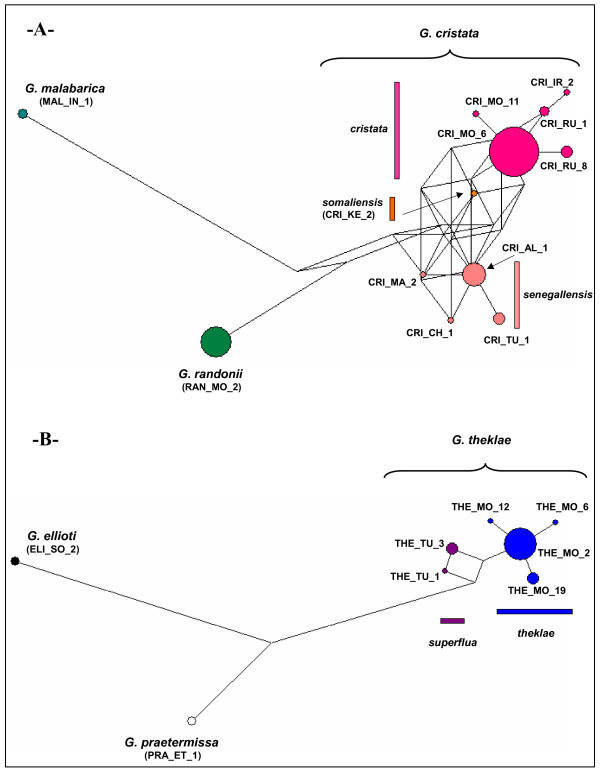
**Median-joining networks**. They were obtained using the short -291 bp- cytochrome b fragment. Each circle represents one haplotype, and its size is proportional to its frequency (see Tables 4–5). Branch length is proportional to number of mutations. 3a) crested lark. 3b) Thekla lark.

Further resolution is revealed by mtDNA data. Within the crested lark complex, the phylogenetic tree revealed three monophyletic groups corresponding to three previously recognized species: *G. randonii *[42], *G. malabarica *and *G. cristata *(Figs. 2, 3a). Lack of resolution for the basal nodes is suggestive of a fairly simultaneous vicariant event for these three species, coincident with the second aridification event at ~1.7 MYA (Table 1, node 6). *G. randonii *and *G. malabarica *show little intraspecific genetic variation, which might be due to their narrow geographic distribution and/or small sample size (Fig. 1a; Table 4). Within the more widely distributed *G. cristata*, three haplotypes cluster with allopatric distribution could be identified (Figs. 1a, 2, 3a) (i) the *cristata *group (monophyletic with average BS = 88; see Table 1, node 8) is by far the most widespread, being present throughout Eurasia (from Korea to Portugal), but also in NE Africa and NW Morocco; (ii) the *senegallensis *group (monophyly poorly supported) is located in the western Sahel and eastern Maghreb; the type specimen of the subspecies *helenae *in the central Sahara clearly belongs to that group (Cri_Al_1 haplotype, see Table 4); (iii) the *somaliensis *group (monophyletic with average BS = 92; Table 1, node 10) is only found in Kenya.

**Table 4:** Phylogeography in the crested lark

	Haplotype												
	
	*G. malabarica*	***G. randonii***	*G. cristata*										
	
			*cristata*					*senegallensis*				*somaliensis*	
**Country**	Mal IN1	**Ran MO2**	Cri MO6	Cri IR2	Cri RU1	Cri MO11	Cri RU8	Cri AL1	Cri CH1	Cri MA2	Cri TU1	Cri KE2	n

Algeria								2					2
Tunisia								5			3		8
Mauritania								1					1
Senegal								2					2
Mali								1		1			2
Niger								1					1
Kenya												1	1
Chad			3						1				4
Morocco		19	23			1							43
Egypte			1										1
S. Arabia			1										1
Israel			1		1								2
Irak			1										1
Iran			5	1									6
Inde	2		1										3
Nepal			1										1
Korea			3										3
Kazakhstan			1										1
Russia			6		1		3						10
Turkey			2										2
France			1										1
Portugal			1										1
n	2	19	51	1	2	1	3	12	1	1	3	1	97

Geographic distribution of haplotypes for the short -291 bp- cytochrome b fragment.

Within the Thekla lark complex, populations currently separated by the Sahara form two highly divergent monophyletic assemblages that diverged concomitantly with the first aridification event at ~2.8 MYA (Fig. 2; Table 1 (node 2) and Table 5). Within *G. theklae *sensu stricto (i.e. the Palearctic lineage), we only found a shallow genetic division among the whole sample (see also Fig. 3b). By contrast, in the Horn of Africa, we found two old mitochondrial lineages, namely *praetermissa *and *ellioti *whose split coincided with the second aridification event at ~1.7 MYA (Table 1, node 3). They correspond to two previously unrecognized evolutionary units. The sequence obtained for a single specimen of the race *hueii *(sampled in the Bale mountains, south of the Rift valley) turned out to be most closely related to *G. praetermissa*, even if it is quite divergent (estimated divergence time: 1.08 MYA, coinciding with the third aridification event; see Table 1, node 4).

**Table 5:** Phylogeography in the Thekla lark

	Haplotype								
	
	*G. theklae*						***praetermissa***	*ellioti*	
	
	*theklae*				*superflua*				
**Country**	The MO2	The MO19	The MO6	The MO12	The TU1	The TU3	**Pra ET1**	Eli SO2	n

France	9	2							11
Morocco	20	2	1	1					24
Algeria	1								1
Tunisia	1				1	4			6
Ethiopia							2		2
Somalia								2	2
n	31	4	1	1	1	4	2	2	46

Geographic distribution of haplotypes for the short -291 bp- cytochrome b fragment.

### Population genetics

#### Population subdivision

In both *G. cristata *and *G. theklae*, none pairwise Φ_ST _value between populations within lineages was significant (all *P *> 0.05 after Bonferroni correction), while as expected, all pairwise Φ_ST _among lineages were significant (Tables 6 and 7).

**Table 6:** Genetic differentiation between populations of *G. cristata*

	E Maghreb (*senegallensis*)	W Sahel (*senegallensis*)	Europe (*cristata*)	Middle East (*cristata*)	Far East (*cristata*)
W Sahel	0.14 ns				
Europe	0.90 ***	0.88 ***			
Middle East	0.89 ***	0.88 ***	0.05 ns		
Far East	0.93 ***	0.93 ***	0.02 ns	0.01 ns	
Morocco (*cristata*)	0.96 ***	0.96 ***	0.14 *	0.15 ns	-0.09 ns

Population pairwise Φ_ST _using Kimura two parameter distance. *P*-values, without Bonferonni correction: ns *P *> 0.10; * *P *< 0.05; ** *P *< 0.01; *** *P *< 0.001.

**Table 7:** Genetic differentiation between populations of *G. theklae*

	France (*theklae*)	Morocco (*theklae*)
Morocco	-0.03 ns	
Tunisia (*superflua*)	0.72 **	0.76 ***

Population pairwise Φ_ST _using Kimura two parameter distance. *P*-values, without Bonferonni correction: ns *P *> 0.10; * *P *< 0.05; ** *P *< 0.01; *** *P *< 0.001.

#### Test of demographic stability

Taken separately, each geographic population of *G. cristata *or *G. theklae *showed non-significant Fu's *Fs *value (all *P *> 0.05), with the exception of Moroccan populations of *G. theklae *(*Fs *= -2.38, *P *= 0.007). Evidence of expansion was found in the *theklae *lineage of *G. theklae *(*Fs *= -2.18, *P *= 0.024) and in the *cristata *lineage of *G. cristata *(*Fs *= -3.71, *P *= 0.003). For the *senegallensis *lineage of *G. cristata*, evidence of expansion was weaker, with nearly significant result (*Fs *= -1.54, *P *= 0.059). Mismatch-distribution analyses were performed for the *theklae*, *cristata *and *senegallensis *lineages. All three distributions fitted the sudden expansion model, as indicated by non-significant tests of goodness of fit (all *P *> 0.10), supporting the hypothesis of a recent expansion in *senegallensis *also. The time to expansion event *t *estimated from mismatch-distribution analyses [95% CI] was *t *~ 38 KYA [0–83] for *senegallensis*, *t *~ 30 KYA [0–64] for *theklae *and *t *~ 22 KYA [0–42] for *cristata*. Because expansions are detected in three distinct lineages that are also the most widespread (see Fig. 1), and because three independent and fairly simultaneous selective sweeps appear unlikely, we feel confident that these patterns reflect demography and not selection [47].

*Test of recurrent gene flow across the Sahara *(divergence between *senegallensis *populations north and south of the Sahara) – Eight independent MDIV runs were conducted and yielded highly consistent results. The data are most consistent with a non-null period of divergence (i.e. *T *> 0; the average mode of *T *was *T *= 0.53 ± 0.13; applying the "standard" calibration of 2%/MY yields *t *~ 29 KYA; 95% CI [2.4–?]), but with complete isolation (M = 0). The latter result is indicated by a strictly decreasing curve for proba(M|data) as long as the prior for T_max _is set < 3, which seems reasonable given the estimated mode of T (see Additional file 4b for a typical run).

### Phenotypic evolution

#### Morphometry

**Figure 4:**
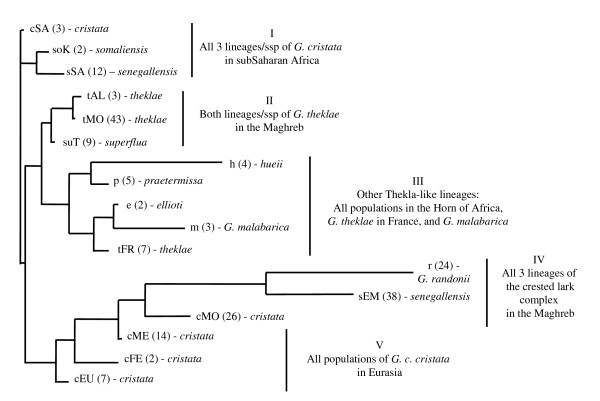
**Morphological tree**. Unrooted NJ tree based on squared Mahalanobis distance, and calculated with six morphological variables. Lower case letter(s) refer to nomenclature for species or genetic groups as defined in Fig. 2 (c = *cristata*; e = *ellioti*; h = *hueii*; m = *G. malabarica*; p = *praetermissa*; r = *G. randonii*; so = *somaliensis*; s = *senegallensis*; su = *superflua*; t = *theklae*); geographic origin is indicated by the following abbreviations (upper case letters: AL = Algeria; EM = Eastern Maghreb; EU = Europe; FE = Far East; FR = France; K = Kenya; ME = Middle East; MO = Morocco; SA = Sahel; T = Tunisia). Numbers in parentheses refer to sample size.

The topology of the tree obtained from the six morphological traits is very different from the genetic one (Shimodaira-Hasegawa test, *P *< 0.001, Figs. 2 and 4). Such a discrepancy can be explained by two factors: (i) taxa do not always cluster according to the species complex they belong to. For instance, *G. malabarica *cluster with members of the Thekla lark complex and not with the more closely related *G. cristata *or *G. randonii*; and (ii) within species complexes, populations tend to cluster on the basis of geographic -and not phylogenetic-affinities, suggesting morphological convergence.

**Figure 5:**
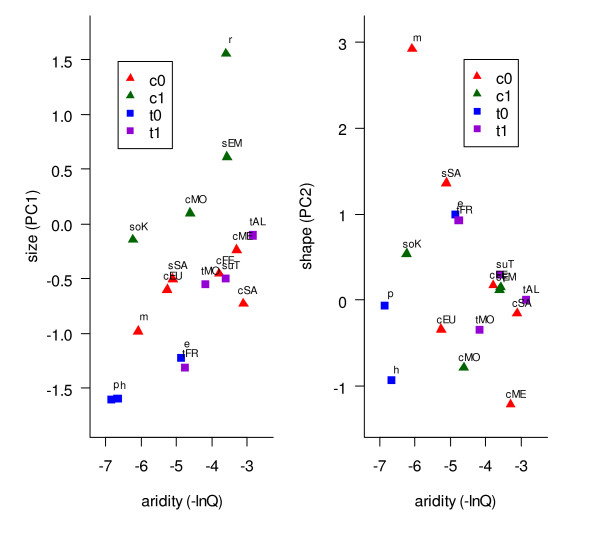
**Role of climate and competition in driving morphological variation**. Relationships between morphology (left: body size; right: body shape, i.e. relative bill width) and environmental aridity for 17 *Galerida *species or populations; legend: c = crested lark; t = Thekla lark; 0 = allopatry; 1 = sympatry (e.g. t1 are populations of the Thekla lark complex that live entirely or mainly in sympatry with a crested lark representative; see Fig. 4 for naming of all populations). Multiple regression analyses suggest that variations in body size (but not in body shape) are influenced by both competition and aridity (see Table 8).

A multiple regression analysis revealed the role of ecological competition and current climate in driving the patterns of body size variation (Table 8, Fig. 5 left). Within both species complexes, populations tended to evolve a larger body size in the presence of a competitor and in more arid areas. However, this latter effect was eclipsed by a significant interaction between aridity and competition (i.e. the trends towards larger body size with increasing aridity is exaggerated in the presence of competition). Generalized Estimating Equations (GEE) were used to control for phylogenetic relationships between populations (see methods). They yielded significant results consistent with ordinary regression (competition: F_1,2 _= 200.41, *P *= 0.0056; interaction between competition and aridity: F_1,2 _= 133.44, *P *= 0.0082). In addition, the significant interaction between species complex and competition factors (see Table 8) suggested that body size shift in the presence of competition affects mainly the crested larks, thus supporting the character displacement hypothesis. However, this latter effect was not found again when the analysis was limited to males (not shown). In contrast with these results for body size, neither competition nor climate explained a significant part of the variation in body shape, as no factor was retained in the multiple regression procedure (Fig. 5 right).

**Table 8:** Role of climate and competition in driving morphological variation

		*Size *(*PC1*)		
*Factor*	*df*	*sgn*(*t*)	*type III F*	*P-value*

species complex (sc)	1	-	5.22	0.043
competition (comp)	1	+	15.36	0.0024
aridity	1	+	2.53	0.14
Interaction (sc × comp)	1	-	6.08	0.031
Interaction (aridity × comp)	1	+	5.49	0.039

ANOVA table presenting factors included in the best regression model for body size (PC1): n = 17 observations; R^2 ^= 0.91; F_5,11 _= 21.23, *P *< 0.0001. For the *sc *and *sc *× *comp *effects, the sign of Student's *t *(sgn(t)) is given here with the Thekla lark complex as a standpoint. For body shape (PC2), no factor was included in the best model.

#### Color variation

In *G. cristata*, the three Chadian light-plumaged *isabellina *specimens shared the most common haplotype of the *cristata *lineage (Cri_MO_6, see Table 4), while the Algerian light-plumaged type specimen of *helenae *had the most common *senegallensis *haplotype (Cri_AL_1). The hypothesis of reciprocal monophyly of *cristata *and *senegallensis *is weakly supported (see *mtDNA Phylogeny *section). Nevertheless, MDIV results suggested that they diverged around 390 KYA (Table 1), seemingly without any subsequent gene flow (at least mtDNA gene flow: MDIV estimate of *M *is 0 as indicated by a strictly decreasing likelihood profile; see Additional file 4c), while the origin of *helenae *and *isabellina *within each lineage is probably much more recent. The *cristata *lineage mostly includes dark-plumaged populations, e.g. in S Europe or Turkey (see Additional file 2 for illustration, and Table 4 for geographic distribution of haplotypes) and the same is true for the *senegallensis *lineage (Additional file 2; Table 4; see also [41] for illustrations of *senegallensis *in sub-Saharan Africa). Hence, each subspecies holds widespread haplotypes of two genetic groups that are mainly made of typical/dark populations. Our molecular results are thus consistent with the hypothesis that several convergence events explain the evolution of plumage color patterns in *G. cristata*.

In *G. theklae*, all three light-plumaged *carolinae *from SE Morocco (Figuig) had haplotypes from the *theklae *haplogroup (two had The_MO_2, one had The_MO_19). By contrast, each light-plumaged *deichleri *of S Tunisia had a distinct haplotype that belonged either to *theklae *(The_MO_2) or to *superflua *haplogroups (The_TU_1 and The_TU_3). Rather than incomplete lineage sorting, MDIV favours a model of recent divergence between populations in Morocco and Tunisia (*t *~ 150 KYA, Table 1), associated with weak but non-null gene flow (*M *~ 0.15, see Additional file 4d). Hence, it is not possible to exclude a single origin of the light colored plumage that characterizes desertic phenotypes in the Thekla lark.

## Discussion

### Support for the climate-driven speciation hypothesis

#### (i) vicariance diversification events coincide with past climatic events

As could be predicted on the basis of traditional systematic hypotheses and results of a previous study [42], both mitochondrial and nuclear DNA data revealed an old (early Pliocene) division between members of two well-supported species groups corresponding to two traditionally recognised species: the Thekla lark and the crested lark.

All subsequent diversification events were remarkably congruent with major late Pliocene and Pleistocene climatic events in Africa, particularly steplike shifts towards more arid conditions near 2.8, 1.7 and 1.0 MYA, and with radiations in co-distributed unrelated species groups [21,23] (see also introduction), bringing support to the climate-driven speciation hypothesis. Periodic expansion of the Sahara appears to have played a major role in cladogenesis, by repeatedly isolating vicariant populations respectively north and south of the main desert landmass (see below), but periods of aridification also impacted populations of the Thekla lark complex living in the Horn of Africa, with (i) a split between populations in highland steppes (*praetermissa*-*hueii*) and xeric grasslands (*ellioti*) that occurred ~1.8 MYA; (ii) the separation of highland steppes populations by the Rift valley dated at ~1.1 MYA.

This scenario should of course be refined in the future and more accurate tests performed. First, the general history of climate change in Africa as described by DeMenocal [21,23] shows local or even regional exceptions [48], which could be taken into account with wider geographical sampling. Perhaps more importantly, the coincidence between divergence and climatic events rely on an assumed cytochrome b mutation rate of 2% per MY, which has not been calibrated for these larks. Although the widespread use of this rate has been criticized [49], there is no alternative to date when no fossil record is available [50], and this rate seems to be largely consistent among avian groups [11], being also relevant in the single songbird clade calibrated to date [16]. In any case, if we except unlikely low values (i.e. less than 1%), all diversification events we report would still be consistent with a late Pliocene or Pleistocene influence, with the possible exception of the Palearctic/Ethiopian split in the Thekla lark (see Table 1). Finally, confidence intervals around estimated values of divergence times are quite large (see Table 1). In the future, a multilocus approach could be used to reduce these confidence limits [17].

Prior to this work, the role of past climatic fluctuations was essentially noticed for savanna-dwelling mammals and forest avian species (see introduction). The present study suggests that the same general pattern also applies for ground-dwelling birds adapted to open habitats (see also [43] for a study of the ostrich *Struthio camelus*). Interestingly, the diversification pattern we describe here for species of steppe-like and montane habitats in and near the tropics is in accordance with the standard model for temperate species [51], but differs from what has been reported for lowland tropical forest species, where most speciation events seem to predate the late Pliocene and Pleistocene epochs [19,20] (for detailed examples in African birds, see [24] – genus *Andropadus*-; [27] – genus *Sheppardia*-; for a counter-example in forest shrews, see [52]). Overall, these results highlight the importance of comparative phylogeographic approaches to unravel the factors underlying diversification processes [53].

#### (ii) current ecological factors shape phenotypic variation

In agreement with previous reports in these taxa (which were based on smaller taxonomic and geographic samplings [42,44]), we found overall a strong discordance between phylogenetic relationships inferred from neutral molecular markers and phenetic similitude among groups based on phenotypic data sets. This suggests a major role for natural selection in driving the patterns of phenotypic evolution in *Galerida *larks, especially given that in birds a strong genetic component has been reported in both morphological traits [54,55] and melanin-based plumage traits [56,57]. However, the exact contribution of phenotypic plasticity cannot be addressed with present data.

#### Morphology

The most striking example concerns morphological convergence in the Maghreb. For the crested lark, convergence is found between *G. randonii *and both *senegallensis *and *cristata *lineages of *G. cristata *(group IV of Fig. 4; see also [44] for previous demonstration of convergence between *G. randonii and senegallensis*). For the Thekla lark, convergence is found between *theklae *and *superflua *(group II of Fig. 4). As the trend in both complexes is for increasing size (Fig. 5 left), this suggested a common, possibly climatic, cause (e.g. James' version of Bergmann's rule, which predicts larger size in more arid areas [46]). However, a multiple regression analysis indicated that variations in body size – such as the trends towards larger body size in the Maghreb- are mainly driven by interspecific competitive interactions between sympatric members of each species complex. Nevertheless, this response to competition is stronger in more arid areas, which is consistent with the view that phenotypic response to natural selection should be strongest during periods or in areas where resources are most limiting [58].

Our analysis also provided some evidence that competition might affect primarily representatives of the crested lark complex, thereby supporting the character displacement hypothesis (significant *species complex *by *competition *interaction in Table 8). Mitochondrial data suggest that in *G. cristata*, the Maghreb was invaded twice (see Guillaumet et al. [44], and discussion below for further details): 1) by the *cristata *lineage, from southern Europe (cEU in Figs. 4 and 5) to the western Maghreb (cMO), and 2) by the *senegallensis *lineage, from sub-Saharan Africa (sSA) to eastern Maghreb (sEM). In presumed source areas (and our corresponding samples, see Additional file 1), *G. cristata *is entirely (sSA) or predominantly (cEU) allopatric with *G. theklae*. Interestingly, the body size of source *G. cristata *populations are strikingly similar to the body size of Maghreban Thekla lark populations that they encountered as they colonized the area (compare cEU *vs *tMO, and sSA *vs *tTU in Fig. 5 left), pointing towards a possible causal mechanism for character displacement. However, we emphasize that further samples will be required to validate this hypothesis (as this effect was lost when the analysis was limited to males).

#### Color

Like in many other bird species [59,60] (but see [61]), and in agreement with a previous report in Morocco [42], plumage variation in *Galerida *larks was in agreement with Gloger's rule (paler plumage in more arid regions, see Additional file 2). Adaptation to desert environments clearly occurred independently in *G. theklae *(e.g. ssp. *deichleri*) and *G. cristata *(e.g. ssp. *helenae*) and probably several times in *G. cristata *although further samples are needed to confirm this. In *G. theklae *on the other hand, a single origin of desert-adapted plumage could not be excluded, as recurrent gene flow was detected among the two desertic populations investigated.

#### Tempo of phenotypic evolution

Our data are suggestive of a punctuated-equilibrium mode of evolution (as suggested in [7] sensu [62]): long periods of relative phenotypic stasis, as might be exemplified by the Thekla-like morphotype in widely divergent phylogenetic lineages (group III of Fig. 4; of course, convergence cannot be ruled out) can sometimes be followed by very rapid episodes of divergence, as a result of e.g. climate-driven adaptation in expanding lineages (morphological convergence in the Maghreb, adaptation of plumage to desertic environment [63,64]). For instance within *senegallensis*, morphological differences between populations north and south of the Sahara, which exhibit only weak and non-significant genetic differentiation (Table 6), are much larger than morphological differences found between valid species that belong to widely divergent species complexes (see Figs. 4, 5).

#### Phenotypic divergence and speciation

Phylogeographic analyses of the Thekla and especially, crested lark complexes reveal a now classical history [65] of multiple fragmentations followed occasionally by the expansion of some refuge lineages that may lead to secondary contact zones between valid or incipient species (Fig. 1). While incidental divergence during periods of isolation may contribute to the speciation process, it is unlikely to play a dominant role here, given the slow accumulation rate of post-zygotic incompatibilities in birds (for instance, fertility loss is generally observed after 5 MY, and rarely before 2 MY [66]). Conversely, rapid phenotypic divergence in response to local ecological conditions as demonstrated here could induce at least partial pre-zygotic isolation at secondary contact zones [1], particularly for such passerine birds for which body size and coloration play an important role in mate choice [5,45]. In the single secondary contact zone studied to date, strong evidence of reproductive isolation was indeed found between *cristata *and *randonii *in Morocco [42,67].

### Role of biogeographic barriers

#### The Sahara

Very few studies have addressed the role of the Sahara in driving speciation events [33] (but see [34]). This is particularly true for the last four million years, a period which is characterized by the onset of arid climatic cycles [35] that might have favoured speciation. Our results suggest three different episodes of north-south vicariance during arid phases of late Pliocene (Palearctic-Ethiopian Thekla split ~2.8 MA), mid-Pleistocene (*G. randonii*-*G. cristata *split ~1.8 MYA) and late Pleistocene (E Maghreb-Sahel split in *senegallensis *~0.029 MYA).

Rather than simple north-south vicariance between *G. cristata *and *G. randonii*, the star-like phylogenetic relationships among the three species of crested larks (Fig. 2) suggests that the divergence between *G. cristata*, *G. randonii *and *G. malabarica *occurred fairly simultaneously ~1.8 MYA. While *G. malabarica *and *G. randonii *are geographically restricted to, respectively, W coastal India and central Maghreb, *G. cristata *is widely distributed in Europe, Asia and Africa (Fig. 1a). However, a sub-Saharan origin is likely for the latter given that the highest genetic diversity is found there (i.e. all three extant genetic lineages that compose it -*cristata*, *senegallensis *and *somaliensis*- are currently found in sub-Saharan Africa, in contrast to all other regions of the world where only one lineage is present; see Fig. 1a). Taken together, these elements suggests a simple speciation history in the crested lark species complex: (i) an ancestral species was distributed in Africa and Asia, occupying part of the current range of the three species *G. cristata*, *G. randonii *and *G. malabarica*; (ii) the expansion of Arabo-Saharan deserts during an arid episode ~1.7 MYA might have led to the formation of three refuge species (i.e. precisely the scenario initially predicted for the Thekla lark complex): *G. malabarica *in India, *G. randonii *in the Maghreb and *G. cristata *in sub-Saharan Africa; (iii) while *G. malabarica *and *G. randonii *would have remained within the limits of their ancient refuge, *G. cristata *would have subsequently expanded its range from its sub-Saharan refuge.

In agreement with a previous report [44], our data are indeed consistent with two independent events of expansion from sub-Saharan Africa in *G. cristata*: (i) the colonization of the whole Eurasia (as well as Morocco secondarily from S Spain) from the eastern Sahel refuge zone (*cristata *lineage); and (ii) the colonization of the eastern Maghreb from the western Sahel refuge zone (*senegallensis *lineage).

A recent range expansion in *cristata *is indicated by significant Fu's *Fs *and the star-like phylogeny of haplotypes (Fig. 3a). Since this taxon is tightly linked with human-modified habitats in parts of its range (particularly, towards west of range [68]), Guillaumet et al. [44] proposed that the *cristata *expansion was facilitated by the development of agriculture that started around ten thousand years ago [69]. When dating this expansion event by mismatch analysis we found a slightly higher but still concordant estimate (22 KYA; 95% confidence interval = 0–42 KYA).

Despite the non-significant (even if marginally so) negative *Fs *value that we found for *senegallensis*, a recent expansion event in this group is suggested by the star-like phylogeny of haplotypes (Fig. 3a) and a mismatch distribution that fitted the sudden expansion model. In fact, it is likely that the signal of expansion has been partly blurred by subsequent isolation and divergence without gene flow between populations N and S of the Sahara, as suggested by MDIV results. We suggest that the Maghreb was colonized from sub-Saharan Africa because *G. cristata *is believed to originate from sub-Saharan Africa (see above). Furthermore, in the case of a recent range expansion, ancestral haplotypes are more likely to be found close to the origin of expansion, whereas derived haplotypes are more likely to be found at the leading edge of range expansion [70]. Such a pattern fits the *senegallensis *haplotype network (as already discussed in [44]): two rare haplotypes found in sub-Saharan Africa tend to occupy an internal position in the network (Cri_CH_1 and Cri_MA_2), while one clearly derived haplotype is only found in the Maghreb (Cri_TU_1; Fig. 3a). However, we emphasize that additional sampling in the Maghreb and sub-Saharan Africa is needed to validate the sub-Saharan origin of the *senegallensis *lineage. Colonisation of the Maghreb may have been favoured by steppe-like vegetation in the Sahara, possibly during the most recent interglacial around 6 KYA [71] (the expansion event for *senegallensis *was dated here at ~38 KYA [0–83]). Then, subsequent aridification of the Sahara led to a contraction of Saharan populations into a few refugia, particularly in mountain areas (e.g. *helenae*), that behave as relic populations (Fig. 1a). This scenario appears compatible with the estimated time of divergence for populations N and S of the Sahara (~29 KYA, with a lower bound of 2.4 KYA; see also Additional file 4) and with two other phylogeographic studies conducted in the lanner falcon *Falco biarmicus *[36] and in the honeybee *Apis mellifera *[37], which also suggest genetic isolation of populations north and south of the Sahara during arid phases [37].

Our results demonstrate that the Sahara did not constitute a permanent barrier throughout geological times. The distribution of *senegallensis *is exemplary, since genetic data still hold the footprints of a recent expansion in the late Pleistocene through the current Sahara, followed by fragmentation and divergence without gene flow between a northern and a southern refuge, as a probable result of increasingly arid conditions (see above). Interestingly, Maghreban populations of *senegallensis *also showed an accelerated rate of morphological evolution [44] (this study). *Galerida *larks thus suggest to us a possible general mechanism by which glacial cycles affecting the Sahara might promote speciation in birds and other taxa (see also [34] for a case of intra-Saharan speciation in *Taterillus *gerbils attributed to climatic cycles).

#### E-W vicariance in the Sahel zone

The star-like phylogeny for the *cristata*, *senegallensis *and *somaliensis *lineages of *G. cristata *(Fig. 2) suggests once again that they result from a single vicariance event that may have occurred around 390 KYA (Table 1). As we discussed above, *G. cristata *seems to originate from sub-Saharan Africa. Accordingly, the divergence between *senegallensis *and *cristata *should correspond to E-W vicariance in the Sahel zone (Fig. 1a), even if we cannot exclude the alternative that one or more lineage only secondarily invaded sub-Saharan Africa.

Interestingly, other E-W vicariance events were also evidenced in the Sahel zone at similar dates in unrelated species such as the hartebeest (*Alcelaphus busephalus*, ~0.39 MYA [7]), and the roan antelope (*Hippotragus equinus*, 0.53–1.02 MYA [28]). It has been suggested that such eastern and western lineages could have been isolated in refuges north and east (resp. north and west) of an expanding central African rainforest belt, during some periods of climate warming [7]. Alternatively, we suggest that refuge populations might instead have been isolated by the lake Chad during such humid phases that led to lake "Mega-Chad" episodes [72].

#### E-W vicariance in the Maghreb

*G. theklae *sensu stricto is structured in two distinct genetic groups (*theklae *and *superflua*) for which the current repartition and divergence date obtained from MDIV analysis suggest a separation in two distinct Maghreban refuges during the penultimate Pleistocene glacial event around 150 KYA (Table 1; for other examples of E-W vicariance in the Maghreb see [73,74]). Since a recent expansion is strongly supported in *theklae *(star-like phylogeny of haplotypes (Fig. 3b), and significantly negative Fu's *Fs*) we find likely that genetic exchanges detected by MDIV took place after the widespread western haplogroup *theklae *underwent an eastwards range expansion ~30 KYA (as indicated by mismatch distribution analysis), thus creating a zone of contact in E Tunisia (see Fig. 1b). However, we cannot totally exclude the possibility that the presence of one *theklae *haplotype in Tunisia (Table 5) reflects incomplete lineage sorting instead of gene flow.

#### The Rift valley

The eastern branch of the Kenyan Rift valley might induce some restrictions to gene flow between sub-Saharan populations of *G. cristata *in the eastern Sahel (*cristata*) and Kenya (*somaliensis*). In addition, our limited data on the former subspecies of *G. theklae *in Ethiopia are indicative of a long-term genetic isolation between populations that live on highland steppes on either side of the Rift valley (*praetermissa*-*hueii *split ~1.1 MYA). Hence, these results agree with the observation that the Rift valley constitutes a major zoogeographical barrier in mammals [75] and birds [76], but also seems to impact the population genetics of species currently distributed on either side (wildebeest *Connochaetes taurinus *[29]; ostrich *Struthio camelus *[43]; Ethiopian wolf *Canis simensis *[75]; African wild dog *Lycaon pictus *[77]).

### Systematics and endemism in Africa

The present study considerably challenges current taxonomic hypotheses in these two species complexes, and paves the way for future revisions.

First, *G. malabarica*, an Indian endemic taxon, turned out to be closely related to crested lark, and not to Thekla lark as previously believed. Hence, apparent disjunction in the distribution of the Thekla lark "superspecies", which comprised three sets of populations separated by Arabo-Saharan deserts (see introduction), proved here to be an artefact of incorrect taxonomy [78]. Interestingly however, the hypothesis of a fragmentation by Arabo-Saharan deserts of a widely distributed ancestral species was actually supported for the crested lark instead (see above).

Secondly, we found a previously unsuspected deep break between Thekla lark populations north and south of the Sahara that occurred ~2.8 MYA. These two sets of populations lack diagnostic morphological (see Fig. 4) as well as plumage color characteristics, which prevented them from being split by previous authors. However, as we have shown that phenotypic convergence is overwhelming in these taxa, it is now clear that phenotypes are not appropriate taxonomic clues. As these two sets of populations are currently separated by a very wide gap of Sahara (Fig. 1b), we believe they should now be recognized as distinct allopatric species.

In addition, the Horn of Africa seems to harbour several narrow-ranged endemic taxa: *praetermissa *appears to be endemic to the high plateaus of Ethiopia north of the Rift valley, *hueii *replaces it in similar habitats south of the Rift valley, while *ellioti *is restricted to arid landscapes in Somalia (Fig. 1b). These allopatric taxa might warrant recognition at the species level, but this conclusion is postponed here because of limited sample size for *ellioti *and *hueii*, and because this study did not include representatives of three other subspecies described from the Horn of Africa (*mallablensis*, *huriensis *and *harrarensis*).

The recognition of new narrow-ranged endemic taxa in the Horn of Africa might significantly supplement the current list of 23 avian species endemic to Ethiopia or Somalia [79]. Interestingly, the only other savanna-dwelling avian species investigated to date, the ostrich, also revealed one previously unexpected endemic species in the Horn of Africa [43], suggesting that more cryptic species remain to be identified in this part of the world. This seems to be quite a general phenomenon in sub-Saharan Africa, where phylogeographical patterns are still poorly understood. Indeed, wherever they are undertaken, molecular phylogenetic studies tend to reveal a more complex biogeographic history than previously recognized, and lead to the recognition of new species (Eastern Arc Mountains [80]; Congo Basin forests [81]; SW Africa [82]). Such findings may have fundamental evolutionary implications for the understanding of large-scale speciation processes, but also for conservation strategies [83] (but see [84]).

## Conclusion

In this study, phylogenetic, population genetics and phenotypic data were integrated in a comparative framework to reconstruct the evolutionary history of two widespread sibling species complexes. In agreement with the climate-driven speciation hypothesis, both past and current climatic factors were found to be influential in driving speciation patterns, as shown respectively by recurrent vicariance mediated by the Sahara, and multiple instances of convergence in morphology (body size) and plumage color.

## Methods

### Specimens

We obtained partial cytochrome b (cyt b) sequences for 154 specimens and complete morphological measurements for 204 specimens (previous morphological and genetic data obtained from earlier studies in Morocco [42] and the Mediterranean region in *G. cristata *[44] were pooled to new material obtained specifically for this study; as a result, we included in our analyses some previously published cyt b sequences: AY165151, AY769740– AY769752, DQ028951– DQ028957). Traditional subspecific classification was used as an indication of plumage variation. Moroccan specimens were mainly collected under license for the present project [42]. Most of the additional specimens consist of museum skins preserved at the French Muséum National d'Histoire Naturelle in Paris, while a few were sent to us directly by contributors and three American museums (see acknowledgements and Additional file 3 for information including voucher/specimen number). Museum vouchers offer a unique opportunity to cover large geographic areas, including currently inaccessible ones (e.g. Somalia). In compensation, however, separate amplification of short DNA fragments had to be devised, which did not preclude from fairly large failures rate depending on whether formaldehyde was used to treat stuffed specimens.

### Mitochondrial DNA data

#### Extraction and sequencing

DNA extraction and sequencing was performed as follows. For fresh tissue samples, the protocol described in Guillaumet et al. [42] was applied (protocol I). Total DNA was isolated using the Sigma GenElute mammalian genomic DNA kit, and a cyt b fragment of 1100 bp was amplified using the primers A (chicken position L-14995) and F (H-16065) [85]. Reactions were performed in 50 μl volumes using 1× buffer, 2.5 mM MgCl_2_, 0.2 mM of dNTPs, 20 pmol of each primer, and 1 unit of Taq DNA polymerase (Promega), at an annealing temperature of 52°C. A 291 bp fragment was then sequenced using primer F as sequencing primer. Amplification products were read with a Pharmacia LKB A.L.F automatic DNA sequencer following recommended procedures.

A specific procedure was applied for museum specimens (protocol II [44] adapted from [86]). First, to avoid contaminations, manipulations were performed in a lab where no fresh samples had previously been handled. Secondly, DNA was extracted from toe pad by using the CTAB procedure [87]. The 291 bp fragment of the cyt *b *was then amplified using primers cyt-H' and F. The amplifications were performed in a final volume of 50 μl. Cycling conditions were 92°C for 40 s, 52°C for 40 s and 72°C for 60 s for 45 cycles. To increase the resolution of weakly supported nodes, we also sequenced longer mtDNA fragments for a subset of specimens of each genetic group identified using the 291 bp alignment. For fresh specimens, this was done by sequencing with both primers A and F. For museum specimens, the cyt b was divided into three contiguous and overlapping fragments: A'C, BD and H'F [44]. After purification (QiaQuick PCR Purification Kit, Qiagena (Holden, Germany)), direct sequencing with the same primers was performed on an automated sequencer following the supplier's procedures (Beckmann Coulter, Inc., Fullerton, CA, USA).

We are confident that cytochrome b sequences obtained were of mitochondrial origin because: (i) there was no stop codon or insertion/deletion in the reading frame, (ii) a stop codon was found at the end of the cyt b gene at the expected position, (iii) no double band was detected on the gels, (iv) the base composition and pattern of base and amino acid substitution were typical of the avian mitochondrial genome (not shown; for additional tests, see [44]).

### Nuclear data

Since mtDNA trees may differ from species trees (e.g. because of stochastic lineage sorting [88]), we attempted to sequence a nuclear gene in representatives of all genetic groups identified with the cyt b (because a large part of museum specimens were fixed with formaldehyde, we had to face a strong failure rate). Two primers designed specifically for this study: bfib1 (5'-TAAAACTATGTATTCTGTTAGTGACAG-3') and bfib2 (5'-TAAACAATTCCTTTATTCATGAATATG-3') allowed us to amplify and sequence 300 bp of the intron 7 of the β-fibrinogen gene (β-fibint 7) in 32 specimens representing all species/taxa covered by mtDNA analyses, with the exception of *hueii *and *malabarica*. The primers were chosen to amplify the region with highest estimated mutation rate (as measured by substitution rate for *G. theklae*-*G. cristata *comparisons in Morocco). Another primer bfib3 (5'-CTTACTGTCCTCAGCACTG-3') was designed and substitute to bfib2 to sequence the most variable 115 bp of the same fragment in four additional specimens of *G. malabarica*. The sequences have been deposited in GenBank (see Tables 2, 3 for Accession Nos).

### Mitochondrial DNA phylogeny

Phylogenetic relationships between haplotypes were inferred using Neighbor-Joining (NJ), Maximum Parsimony (MP), and Maximum Likelihood (ML) methods using Phylowin [89]. One sequence of *Galerida magnirostris *(GenBank AY165169) and *Spizocorys sclateri *(AY165170) were used as outgroups. Pairwise genetic distances were calculated with Kimura two parameter model [90], as preliminary analyses using HKY+Gamma, the best substitution model as identified by ModelGenerator [91] yielded identical results. For ML, a transition over transversion ratio of 3.4 (corresponding to the estimated value for the genus *Galerida*) was applied. The ML tree building algorithm in Phylowin is that of the FASTDNAML program [92]. We also performed Bayesian analyses using MrBayes [93]. However, we obtained identical results with this method and thus only present the results obtained with Phylowin.

We first performed NJ, MP and ML for the short (291 bp) fragment which we obtained for 143 specimens (out of 154), resulting in a total of 20 haplotypes. Bootstrap tests [94] with 5000 replicates for NJ (500 for MP, 100 for ML) were performed to assess the robustness of the clades (i.e. bootstrap support: BS). Since the long fragment could not be obtained in all museum specimens, we had to work with haplotypes of heterogeneous length (31 were detected overall, see Additional file 1a). As a result, the topology (shown in Fig. 2) was obtained with NJ using the pairwise gap removal option (average length = 665 bp). For ML and MP, bootstrap support for a given node was instead obtained using a judiciously chosen subset of haplotypes of the same length (see Additional file 1b). Because the results obtained with the short fragment were nearly identical to those obtained using the longest available alignment, we only present the latter here (Fig. 2), but the results obtained with the short fragment can be seen in the Additional file 1. A combined analysis including β-fibrinogen data did not reveal further resolution for any node and is thus not presented here.

Finally, because assumptions of phylogenetic methods are often violated with intraspecific data sets [95], major groups and alternative potential evolutionary paths among haplotypes were identified for the short fragment using two median-joining networks (the crested and Thekla lark complexes were treated separately) with the parameter ε set to 1 [96].

### Divergence times

Divergence times were estimated using a Bayesian MCMC (Markov Chain Monte Carlo) method as implemented in the program BEAST [97] that simultaneously estimates phylogeny and allows relaxed molecular clocks. Relative rate tests [98] performed with MEGA 3 [99] revealed that a clock-like mode of sequence evolution could not be rejected (all *P *> 0.09), except for the *hueii *sequence as compared to *G. praetermissa *(*P *< 0.05). As a result, we used a constant rate of sequence evolution throughout the tree (CLOC procedure), except for the divergence between *hueii *and *praetermissa *for which uncorrelated rates among branches were allowed (UCLN procedure). Because of heterogeneous length of sequences, different subsets of sequences were used to estimate the age of all nodes (see additional file 1b). For the longest available alignment (1011 bp), the rate of the clock was fixed at 0.01 average substitution per site per million years (according to the standard calibration of 2% sequence divergence per MY in birds [11,16,100]). For shorter alignments, this rate was adjusted to account for slight variations along the sequence (this resulted in rates varying from 0.0085 to 0.0121). The analyses relied on 2.000.000 steps following a discarded burn-in of 100.000 steps. Convergence of the chain to the stationary distribution was confirmed using the application Tracer 1.2 [97]. For each node, we obtained an estimate of the divergence time (mean posterior of their age in years) and the 95% HPD interval for the divergence time estimates (highest posterior density, hereafter confidence interval).

Since BEAST does not correct for intra-specific polymorphism (i.e. it estimates the age of the most recent common ancestor of the genes, not of the populations they are sampled from), divergence times between pairs of recently diverged and/or highly polymorphic taxa were instead estimated using a coalescent-based method (MDIV [101]) that also accounts for recurrent gene flow in the estimation of the divergence time. Several independent runs were performed to ensure that we obtained a good convergence of the chains (we applied a burn-in time of 500.000 steps with 4.500.000 additional steps to estimate the posterior distribution) and to assess the influence of priors on parameter estimates (T_max _and M_max_were allowed to vary from 1 to 10). Divergence within *G. cristata *was estimated as the divergence between the *senegallensis *and *cristata *lineages (see results section and Fig. 2), while divergence within *G. theklae *was estimated as the divergence between the populations in France and Morocco (*theklae *lineage) and populations in Tunisia (*superflua*). Note that upper bounds for credibility intervals around estimated values are not given (this is indicated by a question mark) since they critically depended on an assumed prior for T_max _(as a result of a smooth decrease of proba(T|data) after the mode of T; see Additional file 4c,d).

### Population genetics

The following analyses are based on mtDNA sequences.

#### Test of population subdivision

As regards the two species with large geographic repartition (*G. theklae *and *G. cristata *sensu stricto), we examined geographic differentiation using the short alignment (291 bp). Populations were grouped according to phylogeny (after Fig. 2) and geography, resulting in two groups for each species (see Additional file 3 for detailed repartition of specimens). In *G. theklae*, *theklae *grouped populations from France and Morocco, while *superflua *only consisted of Tunisian samples. In *G. cristata*, *cristata *consisted of four populations (Morocco, Europe, Middle East and Far East), and *senegallensis *of two populations (Sahel and eastern Maghreb; note that Sahel here only encompasses western Sahel, east to W Chad – see Fig. 1a). Genetic differentiation among populations was tested using an F_ST _approach, based on either haplotypic frequencies (conventional approach) or Kimura two parameter genetic distance (Φ_ST _approach), using the program Arlequin 2.0 [102]. Since both methods yielded identical results, we only present the latter. The null distribution of pairwise Φ_ST _values is obtained by randomly permuting haplotypes between populations (analyses presented here are based on 1000 permutations). The *P*-value of the test is the proportion of permutations leading to a Φ_ST _value larger or equal to the observed one.

#### Test of demographic stability

Demographic stability (or selective neutrality) was tested for populations and phylogenetic groupings of populations in *G. theklae *and *G. cristata *(see above) using Fu's *Fs *statistic [103]. When *Fs *was significantly negative (as tested by the permutation procedure implemented in Arlequin 2.0 [102]), we further tested whether observed data fitted a sudden expansion model using a test of goodness of fit (based on SSD statistic) derived from the mismatch-distribution analysis [104] as implemented in Arlequin 2.0. In addition, we calculated the time elapsed since the putative expansion event *t *using the parameter *τ *inferred from the mismatch distribution, using the formula t = τ/2 μ, where *μ *is the mutation rate per gene (taken to be 2%/MY times the length of sequences used).

#### Test of recurrent gene flow across the Sahara

To test if the Sahara acts as a current barrier to gene flow in the *senegallensis *group of the crested lark, we simultaneously estimated the divergence time and the amount of recurrent gene flow using MDIV [101] (see above for details).

### Phenotypic evolution

#### Morphometry

Adult specimens were measured by a single author (AG) for six morphological variables using a caliper (to the nearest 0.1 mm) or a ruler. Measured variables were bill length (bl), bill depth (bd), bill width (bw), tarsus length (tar), tail length (tl) and wing length (wl; see Additional file 3 for details).

The morphological similarity among populations or species was examined by constructing a morphological tree. Only populations with sample size ≥ 2 were considered. To increase sample sizes, both sexes were treated together (similar results were obtained when only males were kept; not shown). The morphological tree was based on Mahalanobis squared distances (which account for the correlation between variables). The resulting matrix was then used to reconstruct a tree using the Neighbor-Joining algorithm. To assess whether morphology accurately reflects history, its topology was then compared to the phylogeny derived from mitochondrial data, using a Shimodaira-Hasegawa test as implemented in R [105]. To make the molecular and morphological trees directly comparable, each morphological group was ascribed a single mtDNA sequence. Because of very low within-group polymorphism, sequence choice is not expected to alter the results, a contention that was checked by performing two distinct trials: 1) we retained the most frequent haplotype in the group considered; 2) one sequence was taken at random. Both approaches yielded identical results.

Since the two trees were incongruent, the possible influences of the climate and interspecific competition on morphology were tested as follows. First, we summarized morphological variation using a principal component analysis (PCA). The first axis of the PCA (hereafter *PC1*) explained 67% of total variance and all six variables were strongly and positively correlated with it (all *r *> 0.63), demonstrating that it is essentially a size axis. The second axis of the PCA (*PC2*) explained 13% of total variance and was a shape axis, distinguishing species or populations with a proportionately wide bill and short tarsus (*r*(PC2,bw) = 0.70; but *r*(PC2,tar) = -0.42).

Then, basic climatic data were obtained from the GHCN (Global Historical Climatology Network) database [106]. Emberger's aridity Index *Q *was computed as a summary climatic statistic: $Q=\frac{2000\cdot P}{M^{2}-m^{2}}$[107], where *P *is the mean annual precipitations in mm, *m *is the mean temperature of the coldest month (used as a proxy of the mean *minimum *temperature of the coldest month) and *M *is the mean temperature of the hottest month (used as a proxy of the mean *maximum *temperature of the hottest month), temperatures being in K. As Q increases non-linearly in more mesic habitat, ln(*Q*) was used instead [108]. Finally, we created the variable *aridity *= -ln(*Q*) so that more arid stations get higher values of the aridity index. Specimens sampled between two stations of the network were ascribed an intermediate value.

To account for interspecific competition, a binary variable called *comp *was created. For each specimen, *comp *equals 1 when it lives sympatrically with a representative of the other species complex, 0 otherwise. Three simple variables as well as all their interactions were thus used as candidate factors: species complex (variable *sc*, either Thekla or crested), competition and aridity. To avoid pseudo-replication, average value of each factor and both *PC1 *and *PC2 *(dependent variables) were taken for each of the seventeen groups defined for building the morphological tree (Fig. 4), and a stepwise forward multiple regression analysis using the Akaike Information Criterion (AIC) was performed to identify relevant factors involved in morphological variation (the model with lowest AIC was retained as the best model).

Because species and populations within each species complex cannot be regarded as independent data points, we also performed a comparative analysis using Generalized Estimating Equations (GEE), which allows multiple factors to be tested simultaneously while controlling for phylogenetic effects [109]. Because only five phylogenetic degrees of freedom were available, we limited the testing procedure to two effects: (i) competition; and (ii) the role of aridity was tested through its interaction with competition, as suggested by the results of ordinary regression (see results section).

All statistical analyses were performed using STATISTICA version 5 (^© ^StatSoft, Inc), and R version 2.0.1 (^© ^The R Foundation for Statistical Computing).

#### Color variation

To test the hypothesis of independent adaptation to desertic environment in both *G. cristata *and *G. theklae*, short cyt b sequences (291 bp) could be obtained for the following sandy desert subspecies: *helenae *(n = 1; Tassili, Algeria) and *isabellina *(n = 3; Ennedi, Chad) for *G. cristata*, *carolinae *(n = 3; Figuig, Morocco) and *deichleri *(n = 3; S Tunisia) for *G. theklae *(see Fig. 1 and Additional file 2). We consider that the hypothesis is supported if each subspecies belong to a distinct monophyletic genetic group which also includes typical dark subspecies. If reciprocal monophyly cannot be ascertained, the hypothesis will still be regarded as supported if genetic groups diverged well before intra-group radiations leading to sandy and dark subspecies, and if no gene flow (as estimated by MDIV) has occurred between the groups since their divergence.

## Authors' contributions

AG carried out molecular genetic and phenotypic studies, performed the analyses and drafted the manuscript. PAC participated in the design of the study and helped to draft the manuscript. JMP carried out DNA sequencing for Museum samples and helped to draft the manuscript. All authors read and approved the final manuscript.

## Supplementary Material

Additional file 2 Illustration of convergent color patterns. Comparative pictures of color patterns in *G. theklae *(*theklae *and *superflua *haplogroups) and *G. cristata *(*cristata *and *senegallensis *haplogroups). Click here for file

Additional file 4 MDIV analyses. Table summarizing MDIV results and results (figures) for typical runs. Click here for file

Additional file 1 MtDNA phylogeny. Variable sites matrix for the full mtDNA data set, subsets of sequences used to estimate bootstrap support and divergence times, and phylogeny obtained with the short (291 bp) cytochrome b fragment. Click here for file

Additional file 3 Basic data. Basic data for the 316 specimens included in the present study. Click here for file
