# Supplementary material for: Climate-driven diversification in two widespread Galerida larks
Source: BMC Evol Biol. 2008 Jan 29;8:32. doi: 10.1186/1471-2148-8-32 (PMC2275783; doi:10.1186/1471-2148-8-32)

Additional file 2 - Illustration of convergent color patterns

Convergent color patterns in four mitochondrial lineages of *Galerida* larks (see also Fig. 2 for the lineages, and additional file 3 for precisions on specimens below). From the left to the right: I- *G. theklae*: (i) two specimens of the *theklae* group (id =1972-1089, Tunisia ; 1982-1128, France); (ii) two specimens of the *superflua* group (1965-1868, Tunisia ; 1965-1866, Tunisia); II- *G. cristata* (iii) two specimens of the *cristata* group (1961-814, Chad; 1960-1647, Yugoslavia); (iv) two specimens of the *senegallensis* group (1962-178, Algeria; 1965-1830, Tunisia). a) dorsal pattern; b) ventral pattern. In each of the four comparisons, the specimen to the left inhabits arid environment (Emberger's index of aridity *Q* varies from 2 to 20), and the specimen to the right more mesic (sub-humid to humid) zones (*Q* varies from 70 to 232).

A2a)


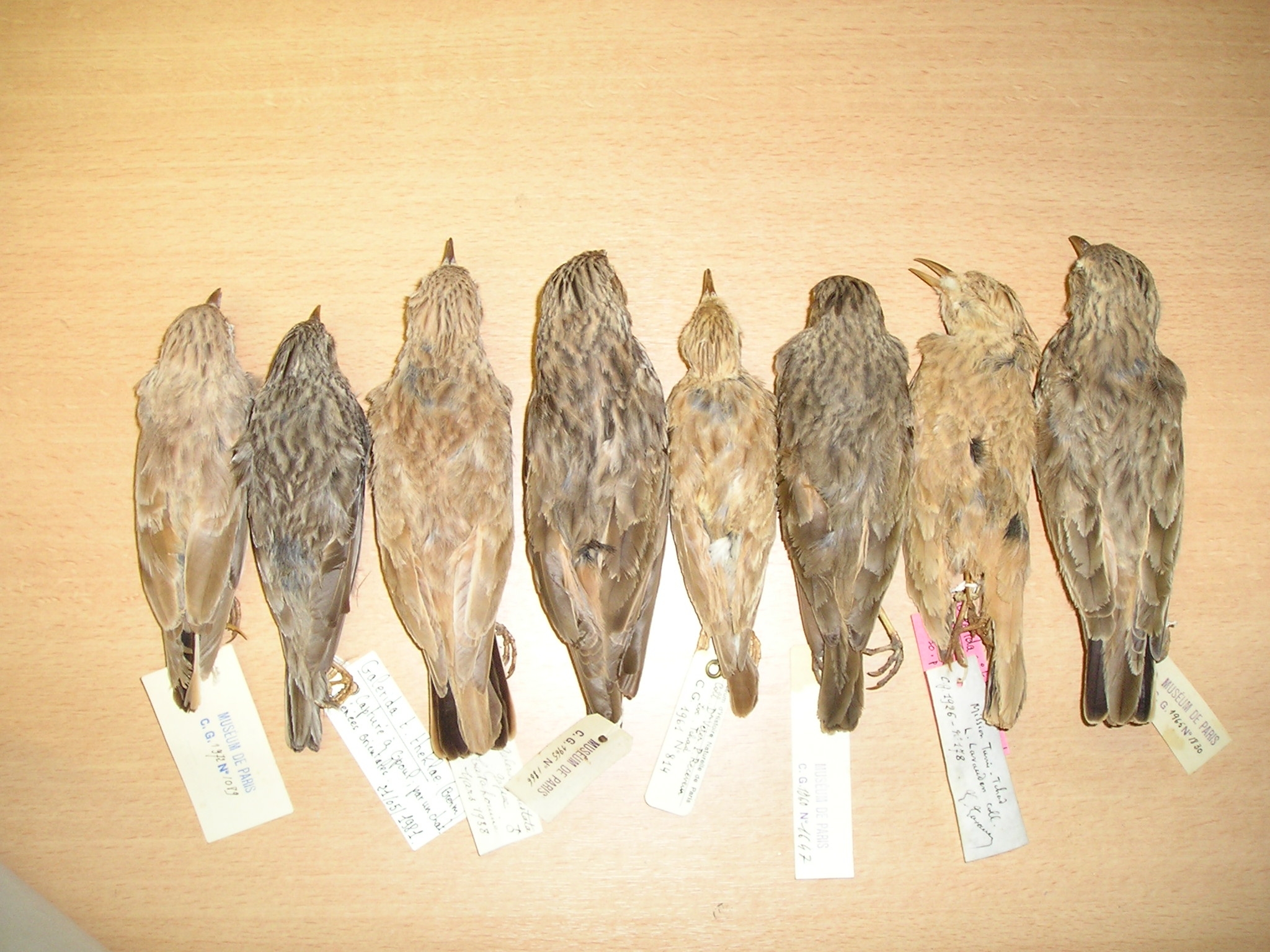


A2b)


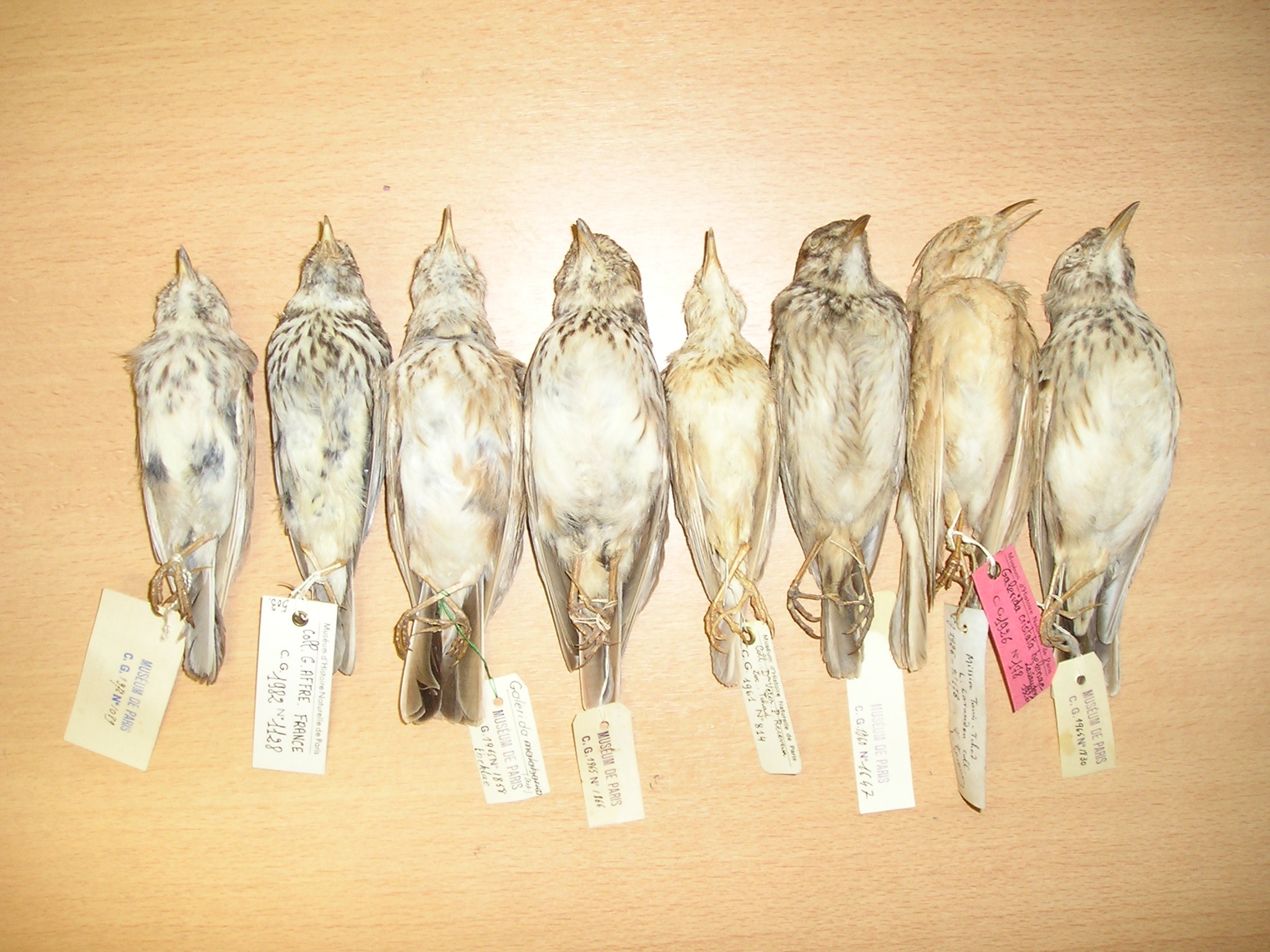

Supplement: Additional file 2 — Illustration of convergent color patterns. Comparative pictures of color patterns in G. theklae (theklae and superflua haplogroups) and G. cristata (cristata and senegallensis haplogroups). [file 1471-2148-8-32-S2.doc]
