# Supplementary material for: Climate-driven diversification in two widespread Galerida larks
Source: BMC Evol Biol. 2008 Jan 29;8:32. doi: 10.1186/1471-2148-8-32 (PMC2275783; doi:10.1186/1471-2148-8-32)
Supplement: Additional file 4 — MDIV analyses. Table summarizing MDIV results and results (figures) for typical runs. [file 1471-2148-8-32-S4.doc]

Additional file 4 - MDIV analyses.

Coalescence-based analyses (MDIV) were performed to study genetic divergence and isolation between three selected pairs of populations.

A4a) Table summarizing MDIV results.

This table gives maximum likelihood value [95 % credibility interval] for theta, effective population size (Nef), scaled migration rate (M), migration rate (m), scaled time of divergence (T), and population divergence in years (t). A mutation rate of 2 % per million years and a generation time of 1 year were used to translate initial scaled parameters into their corresponding estimates. Migration parameters are set to 0 when likelihood surface showed a strictly decreasing profile (see e.g. Fig. A4c, right). Question marks indicate parameter values for which the likelihood surface was too flat to enable inference.

| Comparison | Theta | Nef (×1000) | M | m (×e-06) | T | t |
| --- | --- | --- | --- | --- | --- | --- |
| Sahara  (*senegallensis*: sSA *vs* sEM) | 0.52  [0.19-2.11] | 27.2  [10-110] | 0 | 0 | 0.53 [0.12-?] | 29.3 KYA  [2.4-?] |
|  |  |  |  |  |  |  |
| crested lark  *cristata vs senegallensis* | 1.03  [0.53-2.46] | 58.1  [29.9-138] | 0 | 0 | 3.40 [1.21-?] | 0.39 MYA  [0.07-?] |
|  |  |  |  |  |  |  |
| Thekla lark  *theklae vs superflua* | 0.76  [0.33-2.25] | 42.5  [18.7-126] | 0.15  [0.04-1.77] | 1.72  [0.14-47.3] | 1.79  [0.69-?] | 0.15 MYA  [0.03-?] |

A4b) Sahara

Results for a typical run (priors: T_max_=M_max_=2) indicate a recent but non-null period of divergence (middle, t ~29 KYA), and lack of recurrent gene flow (right).

A4c) crested lark

Results for a typical run (priors: T_max_=10; M_max_=1).

A4d) Thekla lark

Results for a typical run (priors: T_max_=10; M_max_=2).
