# Supplementary material for: Climate-driven diversification in two widespread Galerida larks
Source: BMC Evol Biol. 2008 Jan 29;8:32. doi: 10.1186/1471-2148-8-32 (PMC2275783; doi:10.1186/1471-2148-8-32)
Supplement: Additional file 1 — MtDNA phylogeny. Variable sites matrix for the full mtDNA data set, subsets of sequences used to estimate bootstrap support and divergence times, and phylogeny obtained with the short (291 bp) cytochrome b fragment. [file 1471-2148-8-32-S1.doc]

Additional file 1 - MtDNA phylogeny

(a) Variable sites matrix for the 31 haplotypes found in a 1029 bp fragment of the cytochrome b in the 154 specimens of crested and Thekla lark used in this study (only 30 were used to build Fig. 2, as Cri_KE_2 shows no overlap with a fraction of other haplotypes). (b) Subsets of sequences used to estimate bootstrap support (MP and ML) and divergence times (results in Table 2). (c) Phylogenetic tree obtained for the short (291 bp) cytochrome b fragment. The topology was obtained with NJ; bootstrap support for supported nodes (at least one bootstrap value > 70) is given for NJ (left), MP (right) and ML (below). The number in parentheses refer to the number of haplotypes.

A1a)

111111111111122222222222333333334444444444444455555555555666

11346777899001123344688901122355668012333660122245555666901267899999000

45936258106234735847503311302225175357679092136971689259516976714789168

The_MO_2 TTAGTATCTCTAATTACTCATCCACCTGGAATATCATCGTCCGCTTCCTCTCAACTAGAAACCTACATCCT

The_MO_3 -----------------------------------------..............C...............

The_MO_6 -----------------------------------------..............................

The_MO_12 -----------------------------------------..............................

The_MO_19 .......................................................................

The_MO_26 -----------------------------------------...........................A..

The_TU_1 ...........................A...........................................

The_TU_3 -----------------------------------------------........t...............

The_TU_5 -----------------------------------------------------------------------

Pra_ET_1 .C..C...CA...CCG..T.....T.C.A.G....GC.A..T..CC.TC.C..C.C.AG......T....C

Hue_ET_1 .C..C.C.CA...CCG.CT.......C.A.G....GC.A.....CC.NC.C..CTC.AGG.....A....C

Eli_SO_2 .C....C..AC..CC...T..TT.T.....G...T.C.A.....C..NC....C.C.A.G..T.......C

Cri_MO_6 CCG.CGCTCA..GCC.AC..C..C.TA.AG.CGC.GA..C..ATCC..A.CT...C.A.GG..C...C.TC

Cri_MO_11 -----------------------------------------.ATCC..A.CT...C.A.GG..C...C.TC

Cri_MO_15 ----------------------------------..A..C..ATCC..A.CT...C.A.GG..C...C.TC

Cri_IR_1 CCG.CGCTCA..GCC.AC..C..C.TA.AG.CGC.GA..C..ATCC..A.CTG..C.A.GG..C...C.TC

Cri_IR_2 CNG.CGCTCA..GCC.AC..C..C.TA.AG.CGC.GA..C..ATCC..A.CT...C.A.GG..C...C.TC

Cri_KA_1 CCG.CGCTCA..GCC.AC..C..C.TA.AG.CGC.GG..C..ATCC..A.CT...C.A.GG..C...C.TC

Cri_SA_1 CCG.CGCTCA.GGCC.AC..C..C.TA.AG.CGC.GA..C..ATCC..A.CTG..C.A.GG..C...C.TC

Cri_RU_1 -----------------------------------------------------------------------

Cri_RU_8 CCGTCGCTCA..GCC.AC..C..C.TA.AG.CGC.GA..C..ATCC..A.CT...C.A.GG..C...C.TC

Cri_AL_1 ---------------------------------C.GA..C..ATCCT.A.CT...C.A.GG..C..GC.TC

Cri_CH_1 -----------------------------------------------------------------------

Cri_MA_2 ---------------------------------C.GA..C..ATCC..A.CT...C.A.GG..C...C.TC

Cri_TU_1 -----------------------------------------------------------------------

Cri_TU_5 -----------------------------------------------.A.CT...C.A.GG..C...C.TC

Cri_KE_1 CCG.CGCTCA..GCC.AC..C..C.TAAAG.CGC.GAT.C..ATCC..A.CT...C.A.GG..C...C.TC

Cri_KE_2 CCG.CGCTCA..GCC.AC..C..C.TAAAG.CGC.GAT.C..ATCC..A.CT...C.A.GG..C...C.TC

Ran_MO_2 .CG.CGCTCA..GCC.AC.GC....TA.AG.CGC.GA.ACT.ATCCT.CTCT...C.A.GGT.C...C.TC

Ran_MO_17 .CG.CGCTCA..GCC.AC.GC....TA.AG.CGC.GA.ACT..TCCT.CTCT...C.A.GGT.C...C.TC

Mal_IN_1 --------------------------------------------------------GA.GG..CG.GC..C

1

6666666666666666667777777777778888888888888888899999999999999999999990

0112233344566778891122234455780000112245666778900111222233344457789992

9251706958439581494736981739161457095839489098736278247924625675810356

The_MO_2 CCCATCCGCACGCACACACTCCTCCTACTACACTACGATCCGCCTATAGATCCCGTTACCACACATCCTC

The_MO_3 ......................................................................

The_MO_6 .....................T................................................

The_MO_12 ....................................A.................................

The_MO_19 ..................................................C...................

The_MO_26 ......................................................................

The_TU_1 ............................................C.................C.G.....

The_TU_3 ............................................C.................C.......

The_TU_5 -------------------.........................C.................C......T

Pra_ET_1 .T..CTAA.....GT.........TC....A..C....C..A......A....TA...T..TC.G..T..

Hue_ET_1 ....CTAA.G..T.........C.TC.......C....--------------------------------

Eli_SO_2 .T..CT..T.............CTTC....A..C.TAC..TA...G..A.....A......TC.G...C-

Cri_MO_6 T..CCT.C...........CT.C..CG.CC.GTC..AC...ATGC.C..G....A.C...G.CTGCT...

Cri_MO_11 T..CCT.C...........CT.C..CG.CC.GTC..AC...ATGC.C..G....AAC...G.CTGCT...

Cri_MO_15 T..CCT.C...........CT.C..CG.CC.GTC..AC...ATGC.C..G....A.C...G.CTGCT...

Cri_IR_1 T..CCT.C...........CT.C..CG.CC.GTC..AC...ATGC.C..G....A.C...G.CTGCT..-

Cri_IR_2 T..CCT.C...........CT.C..CG.CC.GTC..AC...ATGC.C..G..T.A.CG..G.CTGCT..-

Cri_KA_1 T..CCT.C...........CT.C..CG.CC.GTC..AC...ATGC.C..G....A.C...G.CTGCT..-

Cri_SA_1 T..CCT.C...........CT.C..CG.CC.GTC..AC...ATGC.C..G....A.C...G.CTGCT..-

Cri_RU_1 -------------------CT.C..CG.CC.GTC..AC...ATGC.C..G..T.A.C...G.CTGCT...

Cri_RU_8 T..CCT.C...........CT.C..CGTCC.GTC..AC...ATGC.C..G....A.C...G.CTGCT...

Cri_AL_1 T..CCT.C...........CT.C..CG.CC...C..AC...ATGC.C..G....A.C...G.TT.CT...

Cri_CH_1 -------------------CT.C..CG.CC.C.C..AC...ATGC.C..G....A.C...G.TT.CT...

Cri_MA_2 T..CCT.C...........CT.C..CG.CC...C..AC...ATGC.C..G....A.C.....TT.CT...

Cri_TU_1 -------------------CT.C..CG.CC...C..AC...ATGC.C..G....A.C..AG.TT.CT...

Cri_TU_5 T..CCT.C...........CT.C..CG.CC...C..AC...ATGC.C..G....A.C...G.TT.CT...

Cri_KE_1 T.TCCT.C.........G. --------------------------------------------------

Cri_KE_2 T.TCCT.C..T......G.CT.C..CG.CC...C..AC...ATGC.C..G..T.A.C...G.TTGCT...

Ran_MO_2 ...C.T.T.......GT...T.C..CG.CC..TCG.AC...ATG..C.......A.C.....TTGCT.C.

Ran_MO_17 ...C.T.T.......GT...T.C..CG.CC..TCG.AC...ATG..C.......A.C.....TTGCT.C.

Mal_IN_1 ..TC.T.T...A...G..ACT.C.TCG.CC...C..AC.T.ATG..CGAG.A....C.....TTGCT.C.

A1b)

|  | Genbank | Node 1  (1007bp) | Node 2  (1007bp) | Node 3  (1007bp) | Node 4  (815bp) | Node 5  (1007bp) | Node 6  (539bp) | Node 7  (539bp) | Node 8  (755bp) | Node 9  (755bp) | Node 10  (452bp) |
| --- | --- | --- | --- | --- | --- | --- | --- | --- | --- | --- | --- |
| The_MO_2 | AY769740 | x | x | x | x | x | x | x | x | x | x |
| The_MO_3 | AY769744 |  |  |  |  |  | x | x |  |  |  |
| The_MO_6 | AY769742 |  |  |  |  |  | x | x |  |  |  |
| The_MO_12 | AY769743 |  |  |  |  |  | x | x |  |  |  |
| The_MO_19 | AY769741 | x | x | x | x | x | x | x | x | x | x |
| The_MO_26 | AY769745 |  |  |  |  |  | x | x |  |  |  |
| The_TU_1 | EF445418 | x | x | x | x | x | x | x | x | x | x |
| The_TU_3 | EF445419 |  |  |  |  |  | x | x |  |  |  |
| The_TU_5 | EF445420 |  |  |  |  |  |  |  |  |  |  |
| Pra_ET_1 | EF445421 | x | x | x | x | x | x | x | x | x | x |
| Hue_ET_1 | EF445422 |  |  |  | x |  |  |  |  |  | x |
| Eli_SO_2 | EF445423 | x | x | x | x | x | x | x | x | x | x |
| Cri_MO_6 | AY769746 | x | x | x | x | x | x | x | x | x | x |
| Cri_MO_11 | AY769748 |  |  |  |  |  | x | x |  |  |  |
| Cri_MO_15 | AY769747 |  |  |  |  |  | x | x |  |  |  |
| Cri_IR_1 | EF445424 | x | x | x | x | x | x | x | x | x | x |
| Cri_IR_2 | DQ028951 | x | x | x | x | x | x | x | x | x | x |
| Cri_KA_1 | EF445425 | x | x | x | x | x | x | x | x | x | x |
| Cri_SA_1 | AY165151 | x | x | x | x | x | x | x | x | x | x |
| Cri_RU_1 | EF445426 |  |  |  |  |  |  |  |  |  |  |
| Cri_RU_8 | EF445427 | x | x | x | x | x | x | x | x | x | x |
| Cri_AL_1 | DQ028953 |  |  |  |  |  | x | x | x | x | x |
| Cri_CH_1 | DQ028954 |  |  |  |  |  |  |  |  |  |  |
| Cri_MA_2 | DQ028956 |  |  |  |  |  | x | x | x | x | x |
| Cri_TU_1 | DQ028955 |  |  |  |  |  |  |  |  |  |  |
| Cri_TU_5 | DQ028957 |  |  |  |  |  | x | x |  |  |  |
| Cri_KE_1 | EF445428 |  |  |  |  |  |  |  |  |  | x |
| Cri_KE_2 | EF445429 | x | x | x | x | x | x | x | x | x | x |
| Ran_MO_2 | AY769749 | x | x | x | x | x | x | x | x | x | x |
| Ran_MO_17 | AY769750 | x | x | x | x | x | x | x | x | x | x |
| Mal_IN_1 | EF445430 |  |  |  |  |  | x | x |  |  |  |

A1c)
