# Supplementary material for: Climate-driven diversification in two widespread Galerida larks
Source: BMC Evol Biol. 2008 Jan 29;8:32. doi: 10.1186/1471-2148-8-32 (PMC2275783; doi:10.1186/1471-2148-8-32)
Supplement: Additional file 3 — Basic data. Basic data for the 316 specimens included in the present study. [file 1471-2148-8-32-S3.doc]

**Additional file 3** Basic data for the 316 specimens of *Galerida* larks included in the present study. **id** = labelling with Museum numbers (if any), or our own labelling; **country** = country in which the specimen was sampled; **lat** = latitude in °N; **long** = longitude in °E; **location** = sampling locality; **cyt b** = 291 bp cyt b haplotype (see Table 1)**; int =** genotype for Intron 7 of the β-fibrinogen gene (see Table 1); **reg** = classification in 17 taxa or populations (see Figs. 4 and 5); **sex** (m=male, f=female, j=juvenile); **bl**= bill length from bill tip to distal edge of nostrils; **bd** = bill depth at distal edge of nostrils; **bw** = bill width at distal edge of nostrils on the upper mandible; **tar** = tarsus length; **tl** = tail length using the T(R) method (see Svensson 1992); **wl** = wing length using the maximum length method (see Svensson 1992); **ar** = value of the aridity index (=-ln(*Q*), where *Q* is Emberger's Index of aridity; **comp**= binary index that takes the value 1 when the specimen lives sympatrically with a representative of the other species complex, 0 otherwise; **mus** = current location of the voucher: A= American Museum of Natural History; B=MVZ Berkeley; C= Field Museum Chicago; P = Museum d'Histoire Naturelle de Paris, M = our lab in Montpellier (France).

| **id** | **country** | **lat** | **long** | **location** | **cyt b** | **int** | **reg** | **sex** | **bl** | **bd** | **bw** | **tar** | **tl** | **wl** | **ar** | **comp** | **mus** |
| --- | --- | --- | --- | --- | --- | --- | --- | --- | --- | --- | --- | --- | --- | --- | --- | --- | --- |
| 1972-1088 | Algeria | 33,15 | 6,05 | Touggourt (Sahara) | Cri_AL_1 |  | sEM | f | 13,3 | 5,4 | 4,5 | 26,5 | 59,0 | 101,0 | -2,01 | 1 | P |
| 1926-196 | Algeria |  |  | Djelfa |  |  | tAL | f | 11,5 | 5,2 | 4,3 | 25,7 | 58,5 | 99,0 | -3,96 | 1 | P |
| 1956-536 | Algeria |  |  | 27kms N of Djelfa (Saharan Atlas) | The_MO_2 |  | tAL | m | 11,0 |  |  | 26,5 | 65,1 | 105,0 |  | 1 | P |
| 1972-1091 | Algeria |  |  | El Oued |  |  | tAL | m | 11,9 | 5,6 | 5,0 | 27,5 | 66,0 | 106,0 | -2,30 | 1 | P |
| 1972-1093 | Algeria |  |  | debila, Terr. De Tougourt, Sahara |  |  | tAL | m | 11,1 | 5,4 | 4,7 | 24,0 | 65,0 | 104,0 | -2,30 | 1 | P |
| 1926-178 | Algeria |  |  | Fort Polignac (=Illizi) | Cri_AL_1 |  |  | m | 13,8 |  |  | 25,2 | 62,0 | 109,0 |  | 0 | P |
| 1927-2230 | Algeria |  |  | Touggourt |  |  |  |  | 13,2 |  |  |  |  |  |  | 1 | P |
| 1927-2231 | Algeria |  |  | Biskra |  |  |  |  | 13,8 |  |  |  |  |  |  | 1 | P |
| 1954-126 | Chad |  |  | Ito chad NE |  |  | cSA | m | 12,6 | 5,4 | 4,3 | 23,8 | 58,0 | 103,0 |  | 0 | P |
| 1959-91 | Chad |  |  | Tchad, Ennedi | Cri_MO_6 | H | cSA | f | 11,9 | 5,4 | 4,7 | 24,6 | 59,0 | 100,0 | -3,12 | 0 | P |
| 1961-814 | Chad | 17,21 | 21,59 | Ouadi Souala, Fada, Enedi | Cri_MO_6 | H | cSA | m | 11,6 | 5,0 | 4,1 | 25,5 | 57,0 | 100,0 | -3,12 | 0 | P |
| 1963-20 | Chad | 15,80 | 20,77 | oum-chalouba, env 230 kms frontière soudan | Cri_MO_6 |  | cSA | j |  |  |  |  |  |  |  | 0 | P |
| 1939-419 | Chad | 12,12 | 15,03 | fort-lamy (N'djamena, front Cameroun) | Cri_CH_1 |  | sSA | f | 9,8 | 5,4 | 4,5 | 23,3 | 47,0 | 95,0 | -5,25 | 0 | P |
| 1939-420 | Chad |  |  | fort lamy |  |  | sSA | f | 12,7 | 4,8 | 4,4 | 24,9 | 57,0 | 93,0 | -5,25 | 0 | P |
| 782664 | China | 36,10 | 120,34 | Tsingtao, Shan-tung |  |  | cFE | m | 12,9 | 4,7 | 4,8 | 23,4 | 65,0 | 105,0 | -4,52 | 0 | A |
| 782666 | China |  |  | Tsingtao, Shan-tung |  |  | cFE | m | 11,7 | 5,2 | 4,5 |  | 64,0 | 106,0 |  | 0 | A |
| Eg1 | Egypt | 29,30 | 30,83 | Fayoum | Cri_MO_6 |  |  | j |  |  |  |  |  |  |  | 0 |  |
| 1974-2082 | Ethiopia |  |  | 5 km Asella-> Mt Cilalo (2640 m) |  |  | h | f | 10,0 |  |  | 24,4 | 52,0 | 98,0 |  | 0 | P |
| 1974-2083 | Ethiopia | 7,10 | 39,78 | Dinsho (Bale) |  |  | h | f | 9,5 | 4,8 | 4,0 | 24,3 | 52,0 | 95,0 | -6,54 | 0 | P |
| 1974-2085 | Ethiopia | 7,10 | 39,78 | Dinsho (Bale) |  |  | h |  | 10,1 | 4,8 | 4,3 | 23,9 | 50,0 | 103,0 | -6,54 | 0 | P |
| 1974-2086 | Ethiopia | 7,10 | 39,78 | Dinsho (Bale) |  |  | h |  | 10,2 | 4,3 | 4,0 | 24,9 | 50,0 | 100,0 | -6,54 | 0 | P |
| 1974-2087 | Ethiopia |  |  | 5 kms Asella dir Mt Cilalo alt 2640 m |  |  | h | m | 11,1 | 4,7 | 3,9 | 25,4 | 55,0 | 106,0 | -7,04 | 0 | P |
| 1974-2091 | Ethiopia |  |  | 20 kms Addis->Gondar |  |  | p | m | 10,3 | 4,8 | 3,6 | 24,5 |  | 97,0 |  | 0 | P |
| 1974-2093 | Ethiopia |  |  | 20 kms Addis->Gondar |  |  | p | m | 10,2 | 5,0 | 3,9 |  | 55,0 | 101,0 |  | 0 | P |
| 1974-2094 | Ethiopia |  |  | 20 kms Addis->Gondar |  |  | p | m | 10,2 | 4,8 | 3,8 |  | 52,0 | 98,0 |  | 0 | P |
| 1974-2096 | Ethiopia |  |  | 25 kms N de Gondar |  |  | p | m | 9,6 | 5,1 | 4,0 |  | 49,5 | 95,0 |  | 0 | P |
| 1974-2098 | Ethiopia |  |  | 10 kms N Gondar |  |  | p | m | 10,2 | 5,0 | 4,2 | 23,5 | 61,0 | 102,0 | -6,65 | 0 | P |
| 1974-2099 | Ethiopia |  |  | 24 kms W of Ambo | Pra_ET_1 | T1 | p | m | 10,6 | 5,3 | 4,3 | 23,7 | 59,0 | 101,0 |  | 0 | P |
| 1974-2100 | Ethiopia |  |  | 22 kms E Ambo alt 2360 m | Pra_ET_1 | T1 | p | m | 10,4 | 4,5 | 4,1 | 23,5 | 58,0 | 101,0 |  | 0 | P |
| 1974-2103 | Ethiopia |  |  | 30 kms N Addis-Abeba |  |  | p | f | 10,4 | 4,7 | 4,3 | 23,2 | 51,0 | 90,0 | -6,94 | 0 | P |
| 1974-2104 | Ethiopia |  |  | 15 kms N Addis-Abeba->Debre Libanos (2800 m) |  |  | p | f | 11,3 | 5,0 | 4,0 | 23,9 | 53,0 | 96,0 | -6,94 | 0 | P |
| MVZ70224 | Ethiopia |  |  | 320 mi W Dessy, Le Gamba; elevation 10000 ft |  |  | p |  |  |  |  |  |  |  |  | 0 | B |
| 1974-2073 | Ethiopia |  |  | vers Aysha |  |  |  | m | 12,8 | 5,8 | 5,1 |  | 58,0 | 100,0 |  |  | P |
| 1974-2074 | Ethiopia |  |  | env Aysha |  |  |  | m | 13,6 | 5,5 | 4,6 | 24,8 | 59,0 | 106,0 |  |  | P |
| 1974-2075 | Ethiopia |  |  | env Aysha |  |  |  | m | 12,6 | 5,4 | 4,9 | 24,5 | 58,0 | 100,0 |  |  | P |
| 1974-2078 | Ethiopia |  |  | 32 kms de Jijija, rte Degehbur |  |  |  | m | 9,7 | 5,0 | 4,4 | 23,2 | 57,0 | 99,0 |  |  | P |
| 1974-2080 | Ethiopia |  |  | 32 kms de Jijija, rte Degehbur |  |  |  | f | 9,5 | 4,4 | 4,4 | 22,6 | 51,0 | 92,0 |  |  | P |
| 1974-2097 | Ethiopia |  |  | 20 kms SW of Gondar |  |  |  | m | 10,6 | 5,0 | 4,2 |  | 53,5 | 100,0 |  | 0 | P |
| 1960-1645 | France |  |  | dunes de Lampaul Plouarzel 29 |  |  | cEU | m | 11,3 | 5,3 | 4,5 |  | 58,0 | 104,0 |  | 0 | P |
| 1960-1649 | France |  |  | Porspoder, 29 |  |  | cEU | m | 11,9 | 5,1 | 4,7 | 25,3 | 59,0 | 103,0 | -5,91 | 0 | P |
| 1978-1423 | France | 42,82 | 2,60 | maury (po) | Cri_MO_6 |  | cEU | m | 12,5 | 4,8 | 4,6 | 23,5 | 65,0 | 107,0 | -4,76 | 1 | P |
| 1978-1434 | France | 47,14 | -0,29 | Puy-Notre-Dame, Maine & Loire |  |  | cEU | f | 12,1 | 5,4 | 4,5 |  | 58,0 | 97,0 |  | 0 | P |
| 1978-1435 | France | 47,14 | -0,29 | Puy-Notre-Dame, Maine & Loire |  |  | cEU | f | 11,6 | 5,0 | 4,6 | 24,9 | 58,0 | 100,0 | -4,84 | 0 | P |
| 1978-1437 | France | 47,14 | -0,29 | Puy-Notre-Dame, Maine & Loire |  |  | cEU | f | 11,5 | 5,1 | 4,5 | 24,7 | 56,0 | 97,5 | -4,84 | 0 | P |
| 1978-1450 | France |  |  | between Vingrau et tuchan, Aude |  |  | tFR | m | 10,8 | 5,3 | 4,5 | 24,6 | 56,0 | 102,0 | -4,76 | 1 | P |
| 1978-1452 | France |  |  | Corbières, between Rivelsaltes et Vingrau PO |  |  | tFR | m | 10,9 | 5,3 | 4,6 | 22,5 | 56,0 | 101,5 | -4,76 | 1 | P |
| 1978-1453 | France |  |  | Corbières, between Rivelsaltes et Vingrau PO |  |  | tFR | f | 11,0 | 4,9 | 5,0 | 24,5 | 55,0 | 98,0 | -4,76 | 1 | P |
| 1978-1454 | France |  |  | Corbières, between Rivelsaltes et Vingrau PO |  |  | tFR | f | 10,3 | 4,8 | 4,6 | 22,3 | 56,0 | 98,0 | -4,76 | 1 | P |
| 1978-1455 | France |  |  | rivesaltes-vingrau |  |  | tFR | f | 10,4 | 4,7 | 4,5 | 22,8 | 58,0 | 95,0 | -4,76 | 1 | P |
| 1978-1456 | France |  |  | Corbières, between Rivelsaltes et Vingrau PO |  |  | tFR | f | 10,6 | 4,9 | 4,6 | 23,5 | 55,0 | 95,0 | -4,76 | 1 | P |
| 1982-1128 | France |  |  | Opoul | The_MO_2 |  | tFR |  | 9,9 | 4,8 | 4,3 | 24,3 | 52,0 | 95,0 | -4,76 | 1 | P |
| F alpha | France |  |  | Camp Joffre (Rivesaltes) | The_MO_2 |  | tFR |  |  |  |  |  |  |  |  | 1 |  |
| F beta | France |  |  | Camp Joffre (Rivesaltes) | The_MO_2 |  | tFR |  |  |  |  |  |  |  |  | 1 |  |
| F2 | France |  |  |  | The_MO_19 |  | tFR |  |  |  |  |  |  |  |  | 1 |  |
| F3 | France |  |  |  | The_MO_2 |  | tFR |  |  |  |  |  |  |  |  | 1 |  |
| F4 | France |  |  | Camp Joffre (Rivesaltes) | The_MO_2 |  | tFR |  |  |  |  |  |  |  |  | 1 |  |
| F5 | France |  |  | Les Courtiels (Fitou) | The_MO_2 |  | tFR |  |  |  |  |  |  |  |  | 1 |  |
| F8 | France |  |  | La Garrigue (Fitou) | The_MO_2 |  | tFR |  |  |  |  |  |  |  |  | 1 |  |
| Fit 1 | France |  |  | Les Courtiels (Fitou) | The_MO_19 |  | tFR |  |  |  |  |  |  |  |  | 1 |  |
| Riv 1 | France |  |  | Camp Joffre (Rivesaltes) | The_MO_2 |  | tFR |  |  |  |  |  |  |  |  | 1 |  |
| Vil 1 | France |  |  | Plan des Faîtes (Villesèque des Corbières) | The_MO_2 |  | tFR |  |  |  |  |  |  |  |  | 1 |  |
| 216182 | India |  |  |  | Cri_MO_6 |  | cFE |  |  |  |  |  |  |  |  | 0 | C |
| 557826 | India | 35,66 | 74,60 | Kashmir Bunji (4500' ~1372 m) |  |  | cFE | m | 12,7 | 4,9 | 4,5 | 23,9 | 63,5 | 104,0 | -3,06 | 0 | A |
| 557833 | India |  |  | Punjab |  |  | cFE |  | 13,0 | 4,9 |  | 23,7 | 56,0 | 97,0 |  | 0 | A |
| 246493 | India |  |  |  | Mal_IN_1 | M2 | m |  |  |  |  |  |  |  |  | 0 | C |
| 344333 | India |  |  | Biligirirangan Hills ca 5500' (~1677 m) |  | M1 | m | m | 11,4 | 5,4 | 5,3 | 23,3 | 54,0 | 99,0 | -6,25 | 0 | A |
| 344334 | India | 23,95 | 78,85 | Karapur ca 2500' SW (~762 m) |  | M2 | m | m | 10,6 | 5,0 | 5,0 | 22,1 | 55,0 | 96,0 | -5,93 | 0 | A |
| 344335 | India |  |  | Bababudan Hills ca 5000' (~1524 m) | Mal_IN_1 | M1 | m | m | 10,5 | 5,3 | 5,3 | 23,0 | 55,5 | 99,0 | -6,07 | 0 | A |
| 1957-940 | Iran | 37,25 | 55,17 | gombad-e-gabous (N) | Cri_MO_6 |  | cME | m | 13,2 | 4,9 | 4,7 | 24,8 | 73,0 | 109,0 |  | 0 | P |
| 1957-942 | Iran |  |  | Bender-Albas Iran S |  |  | cME | m | 13,2 | 4,9 | 4,5 | 26,8 | 73,0 | 113,0 | -3,10 | 0 | P |
| 1957-945 | Iran | 36,55 | 61,17 | sarraks (ne) | Cri_MO_6 |  | cME | f | 11,8 | 4,9 | 4,1 | 22,5 | 61,0 | 101,0 | -3,49 | 0 | P |
| 1960-2160 | Iran |  |  | 30 kms after Jaroin ? |  |  | cME | f | 12,8 | 5,0 | 4,1 | 24,8 | 62,0 | 101,5 |  | 0 | P |
| 1969-2144 | Iran | 35,27 | 51,43 | 50 kms sud Teheran | Cri_MO_6 |  | cME | m | 13,6 | 5,2 | 4,8 |  | 71,0 |  |  | 0 | P |
| 1969-2145 | Iran |  |  | 50 km S Teheran |  |  | cME | m | 13,3 | 5,2 | 4,7 |  | 68,0 | 111,0 |  | 0 | P |
| 1969-2147 | Iran |  |  | near Char Bertiele Iran S |  |  | cME | m | 13,4 | 5,2 | 4,3 | 26,1 | 65,0 | 107,0 |  | 0 | P |
| 1969-2153 | Iran |  |  | Zaheydan |  |  | cME | m | 13,6 | 5,2 | 4,5 | 25,4 | 68,0 | 113,0 |  | 0 | P |
| 1969-2154 | Iran | 29,77 | 51,57 | 10 kms from Shapoor |  |  | cME | m | 14,0 | 5,4 | 4,6 | 26,2 | 61,0 | 110,0 |  | 0 | P |
| Ir1 | Iran |  |  | mooteh | Cri_MO_6 |  | cME |  |  |  |  |  |  |  |  | 0 |  |
| Ir2 | Iran |  |  | Chah-dul village |  | H | cME |  |  |  |  |  |  |  |  | 0 |  |
| Ir3 | Iran |  |  | Farash-barid | Cri_IR_2 |  | cME |  |  |  |  |  |  |  |  | 0 |  |
| Ir4 | Iran |  |  | Borazjan | Cri_MO_6 |  | cME |  |  |  |  |  |  |  |  | 0 |  |
| 1957-23 | Iraq |  |  | Penjivin | Cri_MO_6 |  | cME | m | 13,9 | 4,9 | 4,2 | 24,8 | 69,0 | 112,0 |  | 0 | P |
| Is1 | Israel |  |  | Eilat | Cri_MO_6 |  | cME |  |  |  |  |  |  |  |  | 0 |  |
| Is2 | Israel |  |  |  | Cri_RU_8 |  | cME |  |  |  |  |  |  |  |  | 0 |  |
| 557065 | Italia |  |  | Grosseto |  |  | cEU | f | 11,7 | 4,9 | 4,1 | 25,1 | 55,0 | 100,0 | -4,81 | 0 | A |
| Kaz1 | Kazakhstan | 44,88 | 75,12 | Almaty Oblysy; Akkol', 10 km S, 45 km W; | Cri_MO_6 |  | cFE |  |  |  |  |  |  |  |  | 0 |  |
| 369772 | Kenya |  |  |  |  |  | soK |  |  |  |  |  |  |  |  | 1 | C |
| 707705 | Kenya |  |  | 30 miles N of Marsabit, NFD Kenya | Cri_KE_2 |  | soK | m | 12,3 | 5,4 | 4,6 | 24,3 | 60,0 | 106,0 | -6,23 | 1 | A |
| 707706 | Kenya |  |  | 30 M N of Marsabit, NFD Kenya |  | H | soK | m | 12,2 | 5,8 | 4,6 | 24,9 | 58,5 | 106,0 | -6,23 | 1 | A |
| 252493 | Korea |  |  |  | Cri_MO_6 |  | cFE |  |  |  |  |  |  |  |  | 0 | C |
| 257031 | Korea |  |  |  | Cri_MO_6 |  | cFE |  |  |  |  |  |  |  |  | 0 | C |
| MVZ 154367 | Korea |  |  |  | Cri_MO_6 |  | cFE |  |  |  |  |  |  |  |  | 0 | B |
| 1962-4006 | Mali |  |  | petel? | Cri_AL_1 |  | sSA | m | 12,2 | 5,1 | 4,6 |  | 61,0 | 104,0 |  | 0 | P |
| 1962-4007 | Mali | 12,65 | -8,00 | bamako | Cri_MA_2 |  | sSA |  | 13,5 | 5,4 | 4,5 |  | 60,0 | 105,0 |  | 0 | P |
| 1979-888 | Mauritania | 20,52 | -13,08 | Atar | Cri_AL_1 |  | sSA | m | 12,8 | 5,4 | 4,8 |  | 61,0 | 104,0 |  | 0 | P |
| 1 | Morocco | 35,03 | -5,85 |  | Cri_MO_6 |  | cMO | f | 11,9 | 5,5 | 4,4 | 26,3 | 60,5 | 100,0 | -5,15 | 1 |  |
| 23 | Morocco | 34,22 | -3,28 |  | Cri_MO_6 |  | cMO | m | 13,5 | 5,0 | 4,3 | 25,4 | 64,5 | 105,0 | -3,85 | 1 |  |
| 24 | Morocco | 34,22 | -3,28 |  |  |  | cMO | m | 13,6 | 5,6 | 4,5 | 25,9 | 67,0 | 109,5 | -3,85 | 1 |  |
| 26 | Morocco | 34,22 | -3,28 |  | Cri_MO_6 | H | cMO | f | 12,8 | 5,4 | 4,3 | 25,9 | 62,5 | 104,8 | -3,85 | 1 |  |
| 33 | Morocco | 33,73 | -5,47 |  | Cri_MO_6 |  | cMO | m | 14,5 | 5,4 | 4,5 | 26,0 | 64,5 | 107,5 | -4,76 | 1 |  |
| 40 | Morocco | 33,75 | -7,16 |  | Cri_MO_6 |  | cMO | m | 13,8 | 5,3 | 4,4 | 27,0 | 64,5 | 105,0 | -5,00 | 1 |  |
| 41 | Morocco | 33,74 | -7,15 |  |  |  | cMO | m | 13,4 | 5,4 | 4,2 | 27,1 | 66,0 | 110,2 | -5,00 | 1 |  |
| 42 | Morocco | 33,74 | -7,15 |  | Cri_MO_6 |  | cMO | m | 14,0 | 5,5 | 4,8 | 27,0 | 64,2 | 109,5 | -5,00 | 1 |  |
| 95 | Morocco | 32,61 | -8,67 |  |  |  | cMO | m | 13,0 | 5,4 | 4,5 | 25,8 | 70,0 | 109,0 | -4,31 | 1 |  |
| 114 | Morocco | 32,79 | -8,96 |  | Cri_MO_6 | H | cMO | m | 11,5 | 5,1 | 4,7 | 26,6 | 65,0 | 107,0 | -5,01 | 1 |  |
| 115 | Morocco | 32,79 | -8,96 |  |  |  | cMO | m | 13,6 | 5,4 | 3,9 | 25,0 | 67,0 | 107,5 | -5,01 | 1 |  |
| 118 | Morocco | 32,79 | -8,96 |  | Cri_MO_6 | H | cMO | m | 13,1 | 5,5 | 4,6 | 26,5 | 62,0 | 105,0 | -5,01 | 1 |  |
| 120 | Morocco | 32,79 | -8,96 |  | Cri_MO_6 |  | cMO | f | 13,8 | 5,4 | 4,1 | 25,0 | 63,0 | 101,0 | -5,01 | 1 |  |
| 143 | Morocco | 30,00 | -9,65 |  | Cri_MO_11 |  | cMO | m | 13,3 | 5,4 | 4,5 | 26,2 | 64,5 | 105,5 | -4,35 | 1 |  |
| 149 | Morocco | 30,00 | -9,65 |  | Cri_MO_6 | H | cMO | m | 13,4 | 5,5 | 4,1 | 25,4 | 64,0 | 105,0 | -4,35 | 1 |  |
| 229 | Morocco | 34,22 | -3,96 |  | Cri_MO_6 |  | cMO | m | 12,3 | 5,2 | 4,3 | 25,6 | 62,0 | 107,0 | -4,87 | 1 |  |
| 230 | Morocco | 34,22 | -4,00 |  | Cri_MO_6 |  | cMO | m | 13,4 | 5,4 | 4,5 | 26,6 | 67,8 | 109,0 | -4,87 | 1 |  |
| 241 | Morocco | 32,73 | -7,56 |  | Cri_MO_6 |  | cMO | f | 12,5 | 5,0 | 4,1 | 24,1 | 62,0 | 102,0 | -4,15 | 1 |  |
| 259 | Morocco | 32,68 | -5,28 |  | Cri_MO_6 | H | cMO | f | 11,5 | 5,2 | 4,6 | 23,8 | 58,0 | 95,0 |  | 1 |  |
| 332 | Morocco | 32,94 | -7,89 |  | Cri_MO_6 |  | cMO | m | 14,2 | 5,3 | 4,3 | 26,0 | 64,0 | 103,0 | -4,35 | 1 |  |
| 351 | Morocco | 32,93 | -8,81 |  | Cri_MO_6 |  | cMO | m | 12,8 | 5,4 | 4,8 | 23,7 | 63,0 | 105,0 | -5,01 | 1 |  |
| 379 | Morocco | 32,65 | -7,36 |  | Cri_MO_6 | H | cMO | m | 14,5 |  |  | 24,3 | 71,0 | 110,0 |  | 1 |  |
| 496 | Morocco | 33,70 | -5,60 |  | Cri_MO_6 |  | cMO | m | 12,7 | 5,4 | 4,5 | 25,2 | 64,0 | 105,0 | -4,76 | 1 |  |
| 615 | Morocco | 34,74 | -1,93 |  | Cri_MO_6 |  | cMO | m | 13,7 | 5,6 | 4,7 | 25,6 | 66,0 | 108,0 | -4,09 | 1 |  |
| 616 | Morocco | 34,74 | -1,93 |  | Cri_MO_6 |  | cMO | m | 13,4 | 5,7 | 4,7 | 25,8 | 71,0 | 108,0 | -4,09 | 1 |  |
| 617 | Morocco | 34,74 | -1,93 |  | Cri_MO_6 |  | cMO | f | 12,8 | 5,4 | 4,4 | 25,8 | 63,0 | 105,0 | -4,09 | 1 |  |
| 2001-1607 | Morocco | 35,80 | -5,82 | Tanger | Cri_MO_6 |  | cMO |  | 12,8 | 5,1 | 4,2 | 22,7 | 56,0 | 96,0 | -5,55 | 1 | P |
| 28 | Morocco | 33,50 | -3,60 |  | Ran_MO_2 | H | r | m | 15,8 | 5,5 | 4,9 | 26,8 | 69,0 | 113,0 |  | 1 |  |
| 29 | Morocco | 31,58 | -4,19 |  | Ran_MO_2 |  | r | f | 15,2 | 5,8 | 4,5 | 25,6 | 64,1 | 103,5 | -2,84 | 1 |  |
| 76 | Morocco | 32,10 | -7,68 |  | Ran_MO_2 |  | r | m | 16,6 | 6,3 | 5,0 | 26,8 | 69,2 | 115,5 | -3,82 | 1 |  |
| 77 | Morocco | 32,13 | -7,63 |  |  |  | r | m | 16,2 | 6,2 | 4,8 | 27,3 | 68,5 | 112,0 | -3,82 | 1 |  |
| 79 | Morocco | 32,10 | -7,67 |  |  |  | r | m | 17,6 | 5,9 | 5,1 | 27,5 | 67,0 | 113,0 | -3,82 | 1 |  |
| 80 | Morocco | 32,13 | -7,62 |  |  |  | r | m | 17,0 | 6,2 | 5,1 | 26,7 | 70,5 | 110,0 | -3,82 | 1 |  |
| 83 | Morocco | 32,26 | -8,32 |  | Ran_MO_2 | H | r | m | 15,9 | 6,3 | 4,5 | 27,6 | 63,5 | 109,0 | -4,47 | 1 |  |
| 92 | Morocco | 32,48 | -8,49 |  | Ran_MO_2 |  | r | m | 14,8 | 5,9 | 4,5 | 26,2 | 68,0 | 113,0 | -4,30 | 1 |  |
| 94 | Morocco | 32,57 | -8,50 |  | Ran_MO_2 |  | r | m | 15,9 | 5,6 | 5,1 | 26,8 | 66,5 | 105,5 | -4,30 | 1 |  |
| 195 | Morocco | 30,20 | -5,65 |  | Ran_MO_2 |  | r | m | 15,3 | 5,9 | 4,8 | 25,8 | 62,0 | 108,0 | -2,69 | 1 |  |
| 196 | Morocco | 29,85 | -5,62 |  | Ran_MO_2 | H | r | m | 15,9 | 6,5 | 5,2 | 27,2 | 71,5 | 115,0 | -2,69 | 1 |  |
| 197 | Morocco | 31,29 | -4,31 |  | Ran_MO_2 | H | r | f | 17,9 | 5,5 | 4,4 | 25,8 | 64,3 | 105,5 | -2,84 | 1 |  |
| 198 | Morocco | 32,26 | -2,40 |  |  |  | r | m | 16,1 | 6,0 | 5,2 | 28,5 | 74,5 | 114,0 | -3,03 | 1 |  |
| 199 | Morocco | 32,26 | -2,40 |  | Ran_MO_2 |  | r | m | 16,6 | 6,2 | 5,4 | 25,5 | 69,0 | 114,0 | -3,03 | 1 |  |
| 215 | Morocco | 32,83 | -2,07 |  | Ran_MO_2 |  | r | f | 16,2 | 6,2 | 4,8 | 27,4 | 67,2 | 107,6 | -3,03 | 1 |  |
| 218 | Morocco | 32,85 | -2,06 |  | Ran_MO_2 |  | r | m | 17,8 | 6,1 | 5,0 | 27,9 | 73,0 | 114,0 | -3,03 | 1 |  |
| 220 | Morocco | 32,94 | -2,04 |  |  |  | r | f | 15,1 | 6,4 | 4,5 | 27,3 | 66,5 | 109,0 | -3,03 | 1 |  |
| 227 | Morocco | 34,03 | -2,52 |  | Ran_MO_2 |  | r | m | 17,5 | 6,0 | 5,4 | 28,0 | 74,0 | 119,0 | -3,94 | 1 |  |
| 295 | Morocco | 32,36 | -6,87 |  | Ran_MO_2 | H | r | m | 16,3 | 5,8 | 5,2 | 27,6 | 70,0 | 112,0 | -4,15 | 1 |  |
| 318 | Morocco | 32,65 | -7,36 |  | Ran_MO_2 |  | r | m | 16,6 | 5,9 | 5,1 | 27,5 | 66,0 | 107,0 | -4,15 | 1 |  |
| 436 | Morocco | 32,66 | -6,40 |  | Ran_MO_2 | H | r | m | 15,4 | 5,8 | 5,5 | 26,2 | 72,0 | 114,0 | -4,36 | 1 |  |
| 439 | Morocco | 32,15 | -7,12 |  | Ran_MO_2 |  | r | f | 15,1 | 5,6 | 4,8 | 25,9 | 66,0 | 110,0 | -4,15 | 1 |  |
| 604 | Morocco | 34,07 | -2,04 |  |  |  | r | m | 17,1 | 6,0 | 5,1 | 26,0 | 73,0 | 113,0 | -3,94 | 1 |  |
| 1963-86 | Morocco | 29,02 | -8,90 | Foum el hassane |  |  | r | m | 17,5 | 5,7 | 4,9 | 26,0 | 67,0 | 111,0 |  | 1 | P |
| 1970-364 | Morocco | 33,75 | -2,03 | 30 kms sud Aïn beni mathar | Ran_MO_2 |  | r | f | 17,3 |  | 5,0 | 26,8 | 67,0 | 107,0 |  | 1 | P |
| 7 | Morocco | 35,10 | -5,48 |  | The_MO_2 |  | tMO | m | 11,6 | 5,0 | 4,5 | 25,6 | 59,0 | 101,0 | -5,15 | 1 |  |
| 8 | Morocco | 35,10 | -5,48 |  |  |  | tMO | f | 10,3 | 4,6 | 4,5 | 26,5 | 57,2 | 97,5 | -5,15 | 1 |  |
| 9 | Morocco | 35,10 | -5,48 |  | The_MO_2 |  | tMO | m | 10,9 | 4,8 | 4,3 | 25,7 | 61,0 | 105,0 | -5,15 | 1 |  |
| 10 | Morocco | 35,10 | -5,48 |  | The_MO_2 |  | tMO | f | 10,1 | 5,0 | 4,2 | 25,4 | 58,0 | 96,0 | -5,15 | 1 |  |
| 13 | Morocco | 34,13 | -2,95 |  | The_MO_2 |  | tMO | f | 10,7 | 5,1 | 4,7 | 25,3 | 62,5 | 99,0 | -3,85 | 1 |  |
| 18 | Morocco | 34,13 | -2,95 |  |  |  | tMO | f | 11,3 | 5,4 | 4,5 | 23,5 | 60,5 | 97,0 | -3,85 | 1 |  |
| 30 | Morocco | 33,18 | -5,06 |  |  |  | tMO | m | 10,3 | 5,0 | 4,5 | 26,7 | 62,5 | 103,0 |  | 1 |  |
| 32 | Morocco | 33,18 | -5,06 |  | The_MO_6 | T1 | tMO | m | 11,0 | 5,5 | 4,7 | 26,8 | 65,5 | 104,5 |  | 1 |  |
| 47 | Morocco | 33,53 | -6,86 |  | The_MO_2 |  | tMO | m | 11,0 | 5,5 | 4,9 | 25,7 | 63,5 | 105,5 | -5,00 | 1 |  |
| 50 | Morocco | 33,58 | -6,93 |  |  |  | tMO | m | 11,0 | 5,3 | 4,5 | 24,8 | 64,8 | 104,0 | -5,00 | 1 |  |
| 56 | Morocco | 31,94 | -7,90 |  | The_MO_2 |  | tMO | m | 12,2 | 5,4 | 4,4 | 24,9 | 64,5 | 103,0 | -3,84 | 1 |  |
| 100 | Morocco | 32,67 | -8,93 |  |  |  | tMO | m | 12,0 | 5,1 | 4,5 | 25,5 | 59,0 | 106,0 | -5,01 | 1 |  |
| 101 | Morocco | 32,67 | -8,93 |  |  |  | tMO | m | 10,7 | 5,1 | 4,8 | 24,9 | 58,0 | 99,0 | -5,01 | 1 |  |
| 102 | Morocco | 32,67 | -8,93 |  | The_MO_2 |  | tMO | f | 10,2 | 4,9 | 4,4 | 25,0 | 56,0 | 95,0 | -5,01 | 1 |  |
| 134 | Morocco | 30,03 | -9,65 |  | The_MO_2 |  | tMO | m | 10,5 | 5,2 | 4,6 | 24,0 | 61,0 | 100,5 | -4,35 | 1 |  |
| 152 | Morocco | 29,92 | -9,54 |  | The_MO_12 | T1 | tMO | m | 10,1 | 5,4 | 4,5 | 23,7 | 61,2 | 99,5 | -4,35 | 1 |  |
| 153 | Morocco | 29,88 | -9,52 |  | The_MO_2 |  | tMO | m | 12,3 | 5,2 | 4,3 | 25,2 | 64,8 | 101,0 | -4,35 | 1 |  |
| 158 | Morocco | 29,09 | -10,30 |  | The_MO_2 |  | tMO | m | 11,1 | 5,0 | 4,3 | 24,9 | 60,5 | 102,0 | -4,15 | 1 |  |
| 166 | Morocco | 29,04 | -10,34 |  |  |  | tMO | f | 10,7 | 4,8 | 4,3 | 23,7 | 57,0 | 93,8 | -4,15 | 1 |  |
| 172 | Morocco | 27,89 | -12,88 |  |  |  | tMO | f | 11,0 | 5,0 | 4,9 | 24,9 | 59,5 | 95,0 |  | 1 |  |
| 175 | Morocco | 27,67 | -12,96 |  |  |  | tMO | m | 11,2 | 5,5 | 4,5 | 22,7 | 60,0 | 97,5 |  | 1 |  |
| 179 | Morocco | 27,47 | -12,99 |  | The_MO_2 |  | tMO | m | 11,0 | 5,3 | 4,8 | 23,5 | 58,0 | 95,0 |  | 1 |  |
| 181 | Morocco | 27,18 | -13,18 |  | The_MO_2 | T1 | tMO | m | 10,7 | 4,7 | 4,4 | 25,6 | 62,0 | 102,0 |  | 1 |  |
| 184 | Morocco | 28,09 | -11,35 |  | The_MO_2 |  | tMO | m | 11,4 | 5,4 | 4,8 | 24,2 | 61,0 | 101,0 | -4,14 | 1 |  |
| 202 | Morocco | 32,13 | -1,19 |  |  |  | tMO | f | 10,6 | 5,1 | 4,1 | 25,6 | 62,0 | 99,5 | -2,85 | 1 |  |
| 203 | Morocco | 32,13 | -1,19 |  |  |  | tMO | m | 10,9 | 5,2 | 4,7 | 26,6 | 68,0 | 105,0 | -2,85 | 1 |  |
| 205 | Morocco | 32,13 | -1,19 |  | The_MO_19 | T1 | tMO | m | 11,9 | 5,6 | 4,4 | 27,5 | 67,0 | 108,5 | -2,85 | 1 |  |
| 206 | Morocco | 32,13 | -1,19 |  | The_MO_2 |  | tMO | f | 11,4 | 5,2 | 4,1 | 26,3 | 62,0 | 98,6 | -2,85 | 1 |  |
| 207 | Morocco | 32,13 | -1,19 |  | The_MO_2 |  | tMO | m | 11,1 | 5,3 | 4,9 | 24,7 | 68,0 | 106,0 | -2,85 | 1 |  |
| 216 | Morocco | 32,85 | -2,06 |  | The_MO_2 |  | tMO | m | 11,3 | 5,2 | 4,0 | 25,2 | 68,0 | 106,0 | -3,03 | 1 |  |
| 221 | Morocco | 33,80 | -1,99 |  | The_MO_19 | T1 | tMO | m | 12,2 | 5,6 | 4,7 | 27,6 | 63,0 | 108,5 | -3,94 | 1 |  |
| 242 | Morocco | 32,73 | -7,53 |  | The_MO_2 |  | tMO | m | 10,7 | 4,9 | 4,2 | 23,0 | 62,0 | 103,0 | -4,15 | 1 |  |
| 260 | Morocco | 32,78 | -4,93 |  | The_MO_2 |  | tMO | m | 11,7 |  |  | 26,0 | 68,0 | 109,0 |  | 1 |  |
| 423 | Morocco | 32,60 | -6,38 |  |  |  | tMO | m | 10,5 | 5,4 | 4,5 | 23,4 | 62,0 | 103,0 | -4,36 | 1 |  |
| 518 | Morocco | 33,46 | -5,23 |  | The_MO_2 | T1 | tMO | m | 12,1 | 5,3 | 4,4 | 24,3 | 70,0 | 107,0 |  | 1 |  |
| 547 | Morocco | 33,67 | -5,55 |  | The_MO_2 |  | tMO | m | 11,0 | 5,0 | 4,6 | 26,6 | 68,0 | 107,0 | -4,76 | 1 |  |
| 1963-534 | Morocco |  |  | Khenifra |  |  | tMO | m | 11,9 | 5,6 | 4,4 | 24,9 | 61,5 | 101,5 |  | 1 | P |
| 1963-73 | Morocco |  |  | Ouninet (?) |  |  | tMO | m | 11,1 | 5,4 | 4,5 | 26,0 | 62,0 | 103,0 |  | 1 | P |
| 1963-75 | Morocco |  |  | atlas central Aguelmous |  |  | tMO | m | 11,6 |  | 4,5 | 26,5 | 58,0 | 98,0 |  | 1 | P |
| 1963-79 | Morocco |  |  | between Tantanne and la mer |  |  | tMO | m | 10,6 | 5,1 | 4,0 | 24,0 | 58,0 | 98,0 | -4,15 | 1 | P |
| 1963-80 | Morocco |  |  | Ouaouizert |  |  | tMO | f | 10,5 | 4,6 | 4,2 | 25,5 | 57,5 | 97,0 |  | 1 | P |
| 1969-81 | Morocco |  |  | Bou jrakarêm (?) |  |  | tMO | f | 10,5 | 5,0 | 4,4 | 24,0 | 60,0 | 99,0 |  | 1 | P |
| 1970-357 | Morocco |  |  | Col de Jerada |  |  | tMO | m | 11,4 | 5,1 | 4,5 | 26,5 | 68,5 | 106,0 |  | 1 | P |
| 1970-359 | Morocco |  |  | Saf-Saf |  |  | tMO | m | 10,8 | 5,3 | 4,5 | 25,6 | 61,5 | 101,0 |  | 1 | P |
| 1970-361 | Morocco |  |  | Fouckal, 30 kms S de Berguent |  |  | tMO | f | 10,5 |  |  | 24,9 | 61,5 | 97,0 |  | 1 | P |
| 1970-362 | Morocco |  |  | Plaine des Angaels |  |  | tMO | f | 10,9 | 5,0 | 4,6 | 27,5 | 59,0 | 97,0 |  | 1 | P |
| 96 | Morocco | 32,62 | -8,68 |  | Ran_MO_2 |  |  | m | 13,0 | 5,3 | 4,8 | 26,4 | 64,5 | 105,2 |  | 1 |  |
| 246 | Morocco | 32,47 | -7,02 |  | Cri_MO_6 |  |  | m | 16,2 | 5,7 | 4,8 | 26,6 | 69,0 | 105,0 |  | 1 |  |
| 275824 | Nepal |  |  |  | Cri_MO_6 |  | cFE |  |  |  |  |  |  |  |  | 1 | C |
| 1933-1994 | Niger |  |  | niger R. rive Ansongo |  |  | sSA | m | 13,4 | 5,7 | 5,4 | 24,9 | 61,0 | 102,0 |  | 1 | P |
| 1966-498 | Niger |  |  | delta central du Niger | Cri_AL_1 |  | sSA | f | 12,4 | 5,6 | 4,9 | 25,0 | 61,0 | 101,0 |  | 1 | P |
| 1979-885 | Portugal | 41,70 | -8,83 | viana-castilo | Cri_MO_6 |  | cEU | m | 13,5 | 5,0 | 4,5 | 24,7 | 64,5 | 107,0 | -6,11 | 0 | P |
| 1960-1652 | Romania |  |  | Pantelimon |  |  | cEU | m | 12,1 |  | 3,7 | 26,2 | 55,0 | 97,0 |  | 0 | P |
| rus1 | Russia |  |  | Astrakhan, 17 km S, 55 km W; | Cri_RU_1 |  | cEU |  |  |  |  |  |  |  |  | 0 |  |
| rus10 | Russia |  |  | Blagoveschenskaya, 11 km W | Cri_RU_8 |  | cEU | f |  |  |  |  |  |  |  | 0 |  |
| rus2 | Russia |  |  | Blagoveschenskaya, 11 km W | Cri_MO_6 |  | cEU |  |  |  |  |  |  |  |  | 0 |  |
| rus3 | Russia |  |  | Blagoveschenskaya, 11 km W | Cri_MO_6 |  | cEU | f |  |  |  |  |  |  |  | 0 |  |
| rus4 | Russia |  |  | Blagoveschenskaya, 11 km W | Cri_MO_6 |  | cEU | f |  |  |  |  |  |  |  | 0 |  |
| rus5 | Russia |  |  | Blagoveschenskaya, 11 km W | Cri_MO_6 |  | cEU | f |  |  |  |  |  |  |  | 0 |  |
| rus6 | Russia |  |  | Blagoveschenskaya, 11 km W | Cri_MO_6 |  | cEU | m |  |  |  |  |  |  |  | 0 |  |
| rus7 | Russia |  |  | Blagoveschenskaya, 11 km W | Cri_MO_6 |  | cEU |  |  |  |  |  |  |  |  | 0 |  |
| rus8 | Russia |  |  | Blagoveschenskaya, 11 km W | Cri_RU_8 |  | cEU | m |  |  |  |  |  |  |  | 0 |  |
| rus9 | Russia |  |  | Blagoveschenskaya, 11 km W | Cri_RU_8 |  | cEU | m |  |  |  |  |  |  |  | 0 |  |
| Sa1 | Saudi Arabia | 21,25 | 41,70 | Turabah | Cri_MO_6 |  | cME |  |  |  |  |  |  |  |  | 0 |  |
| 1966-1088 | Senegal |  |  | M'Boro |  |  | sSA | m | 13,1 |  | 5,0 |  | 62,0 | 105,0 |  | 0 | P |
| 1968-895 | Senegal |  |  |  |  |  | sSA |  | 12,6 | 5,6 | 4,9 | 24,0 | 57,0 | 107,0 |  | 0 | P |
| 1972-1016 | Senegal |  |  | mbors | Cri_AL_1 | H | sSA | m | 12,8 | 5,2 | 4,9 |  | 60,0 | 103,0 |  | 0 | P |
| 1972-1017 | Senegal |  |  | Mbors |  |  | sSA | m | 13,6 |  |  |  | 61,0 | 103,0 |  | 0 | P |
| 1975-1718 | Senegal |  |  | delta du senegal |  |  | sSA | f | 11,9 | 5,4 | 4,6 | 23,4 | 54,0 | 95,0 | -5,00 | 0 | P |
| 1975-1719 | Senegal |  |  | delta du senegal |  |  | sSA | f | 12,3 | 5,2 | 4,9 |  |  |  |  | 0 | P |
| 1981-601 | Senegal |  |  | lac de guiers |  |  | sSA | m | 12,6 | 5,9 | 5,0 | 24,1 | 61,0 | 102,0 | -5,00 | 0 | P |
| 1981-602 | Senegal |  |  | lac de guiers |  |  | sSA | f | 11,0 |  |  | 21,8 |  | 91,0 |  | 0 | P |
| 1981-603 | Senegal |  |  | colonat |  |  | sSA | m | 12,0 | 6,2 | 4,4 | 21,9 | 57,0 | 104,0 |  | 0 | P |
| 1981-604 | Senegal |  |  | lac de guiers |  |  | sSA | m | 12,7 |  | 4,6 | 23,2 | 58,0 |  |  | 0 | P |
| 1981-605 | Senegal |  |  | lac de guiers |  |  | sSA | f | 11,1 |  |  | 24,7 | 57,0 | 95,0 |  | 0 | P |
| 1981-606 | Senegal |  |  | lac de guiers |  |  | sSA | m | 13,4 |  |  | 23,3 | 52,0 | 101,0 |  | 0 | P |
| 1981-607 | Senegal |  |  | lac de guiers |  |  | sSA | m | 12,5 | 5,5 | 4,8 | 24,4 | 60,0 | 103,0 | -5,00 | 0 | P |
| 1981-608 | Senegal |  |  | lac de guiers |  |  | sSA | m | 12,9 | 6,1 | 5,1 | 23,2 | 57,0 | 103,0 | -5,00 | 0 | P |
| 1981-609 | Senegal |  |  | lac de guiers | Cri_AL_1 |  | sSA | m | 13,5 | 5,6 | 5,1 | 24,8 | 65,0 | 105,0 | -5,00 | 0 | P |
| 1983-210 | Senegal |  |  | bandia |  |  | sSA | f | 11,3 | 5,9 | 4,5 | 24,9 | 60,0 | 101,0 | -5,33 | 0 | P |
| 2005-1023 | Senegal |  |  | collec privée : St Louis |  |  | sSA | m | 12,6 | 5,1 | 4,5 |  | 57,0 | 99,0 |  | 0 | P |
| 1964-1455 | Somalia | 5,55 | 46,51 | 20 kms S Dusa Mareb | Eli_SO_2 | T2 | e | f | 9,8 | 5,0 | 4,7 | 25,2 | 54,0 | 95,0 | -4,99 | 0 | P |
| 1974-1600 | Somalia | 7,22 | 48,85 | geriban (=Jirriban) | Eli_SO_2 | T1 | e | m | 10,2 | 5,0 | 4,7 | 23,3 | 56,0 | 100,0 | -4,73 | 0 | P |
| 557635 | Somalia |  |  | Berbera |  |  |  | m | 14,0 | 5,3 | 4,7 |  | 58,0 | 102,0 |  | 0 | A |
| 1901-1797 | Tunisia |  |  | Ben Gardobre S Tun, 18 m |  |  | sEM | m | 15,4 | 5,3 | 4,6 | 25,5 | 63,0 | 107,0 |  | 1 | P |
| 1901-1799 | Tunisia |  |  | Ben Gardobre (+18m) |  |  | sEM | f | 13,6 | 5,0 | 4,2 | 26,5 | 59,5 | 101,0 |  | 1 | P |
| 1920-193 ? | Tunisia |  |  | Env de Mr P. Bédé |  |  | sEM | m | 13,9 | 4,8 | 4,3 | 25,5 | 62,0 | 100,0 |  | 1 | P |
| 1924-12 | Tunisia |  |  | Maknang |  |  | sEM | m | 14,5 | 5,6 | 4,7 |  | 60,0 | 108,0 |  | 1 | P |
| 1924-13 | Tunisia |  |  | Maknang (?) |  |  | sEM | f | 13,7 | 5,3 | 4,9 | 25,5 | 60,0 | 98,0 |  | 1 | P |
| 1924-14 | Tunisia |  |  | Gafsa |  |  | sEM | m | 14,6 | 5,7 | 4,9 | 25,5 | 63,0 | 110,0 | -3,22 | 1 | P |
| 1924-19 | Tunisia | 34,43 | 8,78 | Gafsa |  |  | sEM | f | 13,8 | 5,7 | 5,1 | 27,5 | 69,0 | 115,0 | -3,22 | 1 | P |
| 1927-1729 | Tunisia | 35,12 | 10,77 | between El Djem and Sfax |  |  | sEM | m | 14,7 | 5,1 | 4,2 | 25,4 | 59,5 | 100,0 | -3,72 | 1 | P |
| 1927-1730 | Tunisia | 34,48 | 10,35 | between Mahares and Achichina, SE tuni (O) |  |  | sEM | m | 15,1 | 5,4 | 4,6 | 25,0 | 67,0 | 109,0 | -3,66 | 1 | P |
| 1927-1731 | Tunisia |  |  | between El Djem and Sousse, E Tun |  |  | sEM | m | 13,7 | 5,4 | 4,5 |  | 64,0 | 107,0 |  | 1 | P |
| 1927-1732 | Tunisia | 34,48 | 10,35 | between Mahares and Achichina, SE tuni | Cri_AL_1 |  | sEM | m | 15,9 | 5,8 | 5,2 | 25,6 | 71,0 | 113,0 | -3,66 | 1 | P |
| 1927-1733 | Tunisia |  |  | Achichina plains , E tun (0) |  |  | sEM | m | 15,1 | 5,5 | 4,5 | 26,9 | 66,0 | 109,0 |  | 1 | P |
| 1927-1734 | Tunisia |  |  | between Sfax and Aguareb, Gabes |  |  | sEM | m | 15,2 | 5,5 | 5,0 | 27,0 | 66,5 | 108,0 | -3,66 | 1 | P |
| 1927-1734_b | Tunisia |  |  | plains NW of la Marsa N Tun |  |  | sEM | f | 12,6 | 4,9 | 4,5 |  | 59,5 | 107,0 |  | 1 | P |
| 1927-1735 | Tunisia |  |  | between Sfax and Aguareb, E tunis |  |  | sEM | m | 13,8 | 5,6 | 5,0 | 27,6 | 62,8 | 104,5 | -3,66 | 1 | P |
| 1927-1737 | Tunisia | 34,48 | 10,35 | between Mahares and Achichina, E tuni | Cri_AL_1 | H | sEM | m | 14,5 | 4,9 | 4,7 |  | 68,5 | 109,0 |  | 1 | P |
| 1927-1738 | Tunisia |  |  | between Grombalia and Tunis, N Tun | Cri_TU_1 |  | sEM | m | 12,7 | 5,8 | 4,8 |  | 60,0 | 107,0 |  | 1 | P |
| 1927-1740 | Tunisia |  |  | NW Tunisia |  |  | sEM | f | 14,2 | 5,4 | 4,4 |  | 60,0 | 102,0 |  | 1 | P |
| 1927-1743 | Tunisia | 33,88 | 9,98 | between Gabès and El Hamma |  |  | sEM | m | 15,2 | 5,8 | 4,6 | 27,0 | 63,0 | 107,0 | -3,59 | 1 | P |
| 1927-1744 | Tunisia | 34,43 | 8,78 | Gafsa | Cri_AL_1 |  | sEM | m | 13,1 | 5,0 | 4,4 | 26,7 | 68,0 | 110,0 | -3,22 | 1 | P |
| 1927-1745 | Tunisia | 34,95 | 8,57 | Feriana SW Tun |  |  | sEM | m | 14,2 | 5,5 | 4,9 | 24,5 | 67,5 | 109,5 | -3,75 | 1 | P |
| 1927-1746 | Tunisia |  |  | 2 miles E of Tozeur, SW Tun |  |  | sEM | m | 14,5 |  |  |  |  |  |  | 1 | P |
| 1927-1747 | Tunisia | 34,37 | 8,87 | between Gafsa and El-Guandtar, SW Tun |  |  | sEM | m | 15,1 | 5,5 | 4,9 | 26,7 | 66,0 | 111,0 | -3,22 | 1 | P |
| 1927-1748 | Tunisia | 34,37 | 8,87 | between Gafsa and El-Guandtar, SW Tun |  |  | sEM | f | 12,6 | 5,5 | 4,9 |  | 62,0 | 101,0 |  | 1 | P |
| 1927-2218 | Tunisia |  |  | Tamerza |  |  | sEM | m | 13,7 | 5,2 | 4,9 | 24,5 | 59,0 | 100,0 | -3,22 | 1 | P |
| 1927-2224 | Tunisia |  |  | Kerkennah |  |  | sEM | f | 12,7 |  |  |  |  |  |  | 1 | P |
| 1927-2225 | Tunisia | 34,73 | 11,18 | Kerkenah |  |  | sEM | m | 13,8 | 5,5 | 5,0 | 25,5 | 67,0 | 105,5 | -3,72 | 1 | P |
| 1927-2226 | Tunisia |  |  | Kerkennah |  |  | sEM | f | 12,7 |  |  |  |  |  |  | 1 | P |
| 1927-2227 | Tunisia | 34,73 | 11,18 | Kerkenah |  |  | sEM | f | 14,6 | 5,7 | 4,8 | 26,9 | 63,0 | 106,0 | -3,72 | 1 | P |
| 1927-2228 | Tunisia | 34,73 | 11,18 | Kerkenah |  |  | sEM | f | 13,2 |  | 5,0 | 24,8 | 65,0 | 108,0 |  | 1 | P |
| 1927-2229 | Tunisia | 34,73 | 11,18 | Kerkenah |  |  | sEM | f | 12,9 |  | 4,4 | 24,9 | 61,0 | 101,0 |  | 1 | P |
| 1927-2234 | Tunisia |  |  | Nefta |  |  | sEM | m | 14,6 |  |  |  |  |  |  | 1 | P |
| 1927-2235 | Tunisia |  |  |  |  |  | sEM |  | 13,4 |  |  |  |  |  |  | 1 | P |
| 1927-2236 | Tunisia |  |  | Nefta |  |  | sEM | f | 14,2 |  |  |  |  |  |  | 1 | P |
| 1960-1654 | Tunisia | 34,87 | 9,33 | Mezzouna |  |  | sEM | m | 14,2 | 5,9 | 5,0 | 26,2 | 64,0 | 110,0 | -3,48 | 1 | P |
| 1960-1655 | Tunisia | 34,87 | 9,33 | Mezzouna SW Sfax |  |  | sEM | m | 14,0 | 6,1 | 5,0 |  |  | 106,0 |  | 1 | P |
| 1960-1656 | Tunisia |  |  | djebel cherichera tunisie C |  |  | sEM | f | 13,6 |  | 4,9 | 26,0 | 61,0 | 104,0 |  | 1 | P |
| 1961-83 | Tunisia |  |  | Tozeur |  |  | sEM |  | 13,1 |  |  |  |  |  |  | 1 | P |
| 1965-1818 | Tunisia |  |  | Grombalia |  |  | sEM | m | 13,8 | 5,3 | 4,7 | 25,9 | 65,0 | 109,0 | -4,49 | 1 | P |
| 1965-1819 | Tunisia | 35,40 | 9,55 | Hadjeb el aioun |  |  | sEM | m | 15,4 | 5,5 | 4,5 | 25,7 | 61,0 | 105,0 | -4,02 | 1 | P |
| 1965-1819_b | Tunisia | 32,93 | 10,47 | Tatahouine |  |  | sEM | m | 13,8 | 5,8 | 4,5 | 26,7 | 63,0 | 107,0 | -3,31 | 1 | P |
| 1965-1821 | Tunisia |  |  | Aadjeb-el-aïoun |  |  | sEM | m | 15,5 |  |  |  |  |  |  | 1 | P |
| 1965-1823 | Tunisia |  |  | Aadjeb-el-aïoun |  |  | sEM | m | 13,2 | 5,4 | 4,3 | 25,2 | 60,0 | 101,0 | -4,02 | 1 | P |
| 1965-1824 | Tunisia |  |  | St Germain |  |  | sEM | m | 14,3 | 5,6 | 5,1 | 24,6 | 66,0 | 110,0 | -4,49 | 1 | P |
| 1965-1826 | Tunisia |  |  | Medjez-el-Bab |  |  | sEM | f | 13,2 |  |  |  |  |  |  | 1 | P |
| 1965-1828 | Tunisia |  |  | Potinville |  |  | sEM |  | 13,6 |  |  |  |  |  |  | 1 | P |
| 1965-1830 | Tunisia |  |  | Potinville | Cri_TU_1 |  | sEM |  | 14,9 | 6,0 | 5,1 | 26,5 | 61,0 | 106,0 | -4,49 | 1 | P |
| 1972-1071 | Tunisia | 33,90 | 10,10 | Gabès |  |  | sEM | m | 14,7 | 6,0 | 5,2 | 25,5 | 68,5 | 109,5 | -3,59 | 1 | P |
| 1972-1072 | Tunisia | 34,42 | 8,62 | Redeyef | Cri_AL_1 | H | sEM | m | 13,5 | 5,9 | 4,8 | 25,8 | 64,5 | 107,0 | -3,22 | 1 | P |
| 1972-1073 | Tunisia | 33,90 | 10,10 | Gabes |  |  | sEM | m | 14,8 | 6,1 | 5,2 | 27,0 | 63,0 | 109,0 | -3,59 | 1 | P |
| 1972-1074 | Tunisia | 33,90 | 10,10 | near Gabès |  |  | sEM | m | 14,8 | 6,0 | 5,3 | 25,5 | 65,5 | 110,0 | -3,59 | 1 | P |
| 1972-1075 | Tunisia | 33,90 | 10,10 | Gabès (Oued Zeragh) |  |  | sEM | m | 15,2 | 5,9 | 4,8 | 24,9 | 66,0 | 106,5 | -3,59 | 1 | P |
| 1972-1076 | Tunisia |  |  | Gafsa road, Gabes | Cri_AL_1 |  | sEM | m | 16,9 | 5,9 | 4,8 | 24,7 | 61,0 | 103,0 |  | 1 | P |
| 1972-1077 | Tunisia | 33,90 | 10,10 | Gabès | Cri_TU_1 |  | sEM | m | 15,7 | 5,8 | 5,0 | 25,7 | 64,0 | 109,0 | -3,59 | 1 | P |
| 1972-1078 | Tunisia | 35,12 | 10,75 | Louza, near Djebeniana (40 kms N Sfax) |  |  | sEM | m | 15,4 | 5,5 | 4,8 | 27,0 | 63,0 | 107,0 | -3,72 | 1 | P |
| 1972-1079 | Tunisia | 34,33 | 8,95 | El-Guettar (near Gafsa) |  |  | sEM | m | 14,8 |  | 4,3 | 25,5 | 60,5 | 106,5 |  | 1 | P |
| 1972-1080 | Tunisia | 34,42 | 8,62 | Redeyef |  |  | sEM | m |  |  | 5,0 | 25,5 | 68,5 | 112,0 |  | 1 | P |
| 1972-1081 | Tunisia | 34,42 | 8,62 | Redeyef |  |  | sEM | f | 12,6 | 5,7 | 4,5 |  | 61,0 | 101,0 |  | 1 | P |
| 1972-1082 | Tunisia | 33,90 | 10,10 | Gabès |  |  | sEM | f | 14,2 |  | 4,6 | 25,3 | 59,5 | 101,0 |  | 1 | P |
| 1972-1083 | Tunisia |  |  | Oued Zeragh, gabès |  |  | sEM | f | 15,5 |  |  |  |  |  |  | 1 | P |
| 1972-1086 | Tunisia | 33,90 | 10,10 | Gabès |  |  | sEM | f | 14,1 | 5,2 | 4,4 | 25,2 | 61,0 | 100,0 | -3,59 | 1 | P |
| 1972-1087 | Tunisia | 34,67 | 10,98 | 22 km E Sfax (Aguarelle) |  |  | sEM | f | 13,6 | 5,1 | 3,8 | 25,2 | 59,4 | 100,0 | -3,72 | 1 | P |
| 1972-1746 | Tunisia | 33,93 | 8,13 | 2 miles E of Tozeur |  |  | sEM | m | 14,6 | 5,5 | 5,1 | 27,5 | 65,5 | 109,0 | -2,83 | 1 | P |
| 1927-1750 | Tunisia |  |  | N of la Marsa, N Tuni |  |  | suT | f | 10,5 | 4,9 | 4,6 | 25,0 | 56,0 | 96,0 | -4,49 | 1 | P |
| 1927-1753 | Tunisia |  |  | between Gabès and El Hamma (300m alt) |  |  | suT | f | 10,5 |  | 4,2 | 24,3 | 55,4 | 97,0 |  | 1 | P |
| 1927-2207 | Tunisia |  |  | Tamerza |  |  | suT | f | 10,1 |  | 3,9 | 24,6 | 59,0 | 100,0 |  | 1 | P |
| 1960-1657 | Tunisia |  |  | Mengel-S de Sousse |  |  | suT | f | 10,4 | 4,9 | 4,3 |  | 56,5 | 94,5 |  | 1 | P |
| 1960-1660 | Tunisia |  |  | Kallat fedj er resta | The_TU_3 |  | suT | f | 10,3 | 4,7 | 4,5 | 25,1 | 61,0 | 98,0 |  | 1 | P |
| 1965-1838 | Tunisia |  |  | Hadjeb-Aïoun | The_TU_3 |  | suT | m | 10,7 | 5,0 | 4,2 | 24,0 | 62,0 | 104,0 | -4,02 | 1 | P |
| 1965-1842 | Tunisia |  |  | Potinville-Djebel recas |  |  | suT |  | 10,8 | 5,5 | 5,0 | 24,9 | 61,0 | 105,0 |  | 1 | P |
| 1965-1844 | Tunisia |  |  | Tatahouine |  |  | suT | m | 12,0 | 5,3 | 5,0 | 25,1 | 60,0 | 105,0 | -3,31 | 1 | P |
| 1965-1846 | Tunisia |  |  | Tatahouine |  |  | suT | f | 9,8 | 4,8 |  |  | 59,0 | 96,0 |  | 1 | P |
| 1965-1858 | Tunisia |  |  | Tatahouine | The_TU_1 | T1 | suT | m | 11,0 | 5,3 | 4,6 | 25,6 | 65,0 | 106,0 | -3,31 | 1 | P |
| 1965-1864 | Tunisia |  |  | Tatahouine |  |  | suT | f | 13,2 | 5,5 | 4,7 | 24,6 | 60,0 | 102,0 | -3,31 | 1 | P |
| 1965-1866 | Tunisia |  |  | Djebel-Reces | The_TU_3 |  | suT |  | 11,0 | 5,6 | 4,8 | 24,9 | 65,0 | 108,0 |  | 1 | P |
| 1972-1089 | Tunisia | 34,42 | 8,62 | Redeyef | The_MO_2 | T1 | suT | m | 10,5 | 5,1 | 4,7 | 23,9 | 60,0 | 105,0 | -3,22 | 1 | P |
| 1972-1090 | Tunisia |  |  | Gabès | The_TU_3 | T1 | suT |  |  |  |  |  |  |  |  | 1 | P |
| 1969-2162 | Turkey |  |  | 30 kms from Gorum |  |  | cME | m | 13,4 | 5,5 | 4,4 | 26,0 | 66,0 | 110,0 |  | 0 | P |
| 1969-2163 | Turkey |  |  | lac from Gölbazi |  |  | cME | m | 14,5 | 5,4 | 4,7 | 26,7 | 66,0 | 113,0 |  | 0 | P |
| 1969-2164 | Turkey |  |  | 30 kms from Gorum |  |  | cME | m | 13,1 | 5,4 | 4,6 | 26,7 | 68,0 | 114,0 |  | 0 | P |
| 1969-2165 | Turkey |  |  | 50 kms from Gorum |  |  | cME | m | 14,3 | 5,7 | 4,7 | 26,5 | 62,0 | 111,0 |  | 0 | P |
| 1969-2166 | Turkey |  |  | golbazi | Cri_MO_6 |  | cME | m | 12,3 | 4,8 | 4,0 |  | 70,0 | 112,0 |  | 0 | P |
| 1969-2167 | Turkey |  |  | golbazi | Cri_MO_6 |  | cME | f | 12,9 | 5,0 | 4,4 | 24,5 | 66,0 | 103,0 |  | 0 | P |
| 1969-2168 | Turkey |  |  | lac de Gölbazi |  |  | cME | f | 12,3 | 5,4 | 4,2 | 26,1 | 65,0 | 106,0 |  | 0 | P |
| 1960-1647 | Yugoslavia | 42,42 | 18,77 | cattaro (Kotor) |  |  | cEU | m | 12,5 | 5,1 | 4,0 | 24,9 | 64,0 | 106,0 | -5,55 | 0 | P |
